# Enhancing phenolic and flavonoid recovery from Vietnamese balm using green solvent-based ultrasonic-enzymatic-assisted extraction

**DOI:** 10.1016/j.ultsonch.2025.107546

**Published:** 2025-09-01

**Authors:** Tan Phat Vo, Thi Hoang Trang Nguyen, Ha Bao Tran Nguyen, Hoang Nhan Nguyen, Nguyen Van Nhi Le, Minh Hoa Ha, Gia Bao Pham, Dinh Quan Nguyen

**Affiliations:** a Laboratory of Biofuel and Biomass Research, Faculty of Chemical Engineering, Ho Chi Minh City University of Technology (HCMUT), 268 Ly Thuong Kiet Street, District 10, Ho Chi Minh City, Vietnam; b Vietnam National University Ho Chi Minh City, Linh Trung Ward, Thu Duc City, Ho Chi Minh City, Vietnam; c School of Chemical and Environmental Engineering, International University (HCMIU), Linh Trung Ward, Thu Duc City, Ho Chi Minh City, Vietnam

**Keywords:** Ultrasonic-enzymatic-assisted extraction, Phenolics, Flavonoids, Vietnamese balm, Natural deep eutectic solvents

## Abstract

The research explored the extraction of phenolic and flavonoids from *Elsholtzia ciliata* (Vietnamese balm) using a natural deep eutectic solvent (NADES)-based ultrasonic-enzymatic-assisted extraction (UAE-EAE) technique. A range of NADESs was evaluated to investigate the most suitable solvent system. The NADES synthesized from lactic acid and choline chloride provided the highest recovery of phenolics and flavonoids. Single-factor experiments were conducted to assess how individual process variables influence the recovery of phenolics and flavonoids. Key factors affecting extraction performance were assessed using the Plackett-Burman approach. Optimal conditions were established through the Box-Behnken Design model. The optimal extraction parameters for TPhC were determined as a solid-to-liquid ratio (SLR) of 1:59 g/mL, water content (WC) of 12 %, ultrasonic temperature of 43 °C, ultrasonic time of 6.88 min, and incubation time of 6.33 min. For TFlC, the optimal conditions were established at SLR of 1:67 g/mL, WC of 14 %, and enzyme activity of 1.28 U/g. Under these optimized conditions, UAE-EAE achieved the maximum recovery of total phenolic content (36.41 ± 2.50 mg GAE/g) and total flavonoid content (18.74 ± 1.28 mg QE/g). A second-order kinetic model was adopted to analyze the extraction mechanism. Structural and surface changes in the plant matrix before and after extraction were further investigated using scanning electron microscopy (SEM) and X-ray diffraction (XRD). This method demonstrates high efficiency and eco-friendliness in extracting plant-derived bioactive compounds using NADES-based UAE-EAE.

## Graphical abstract

**Figure:**
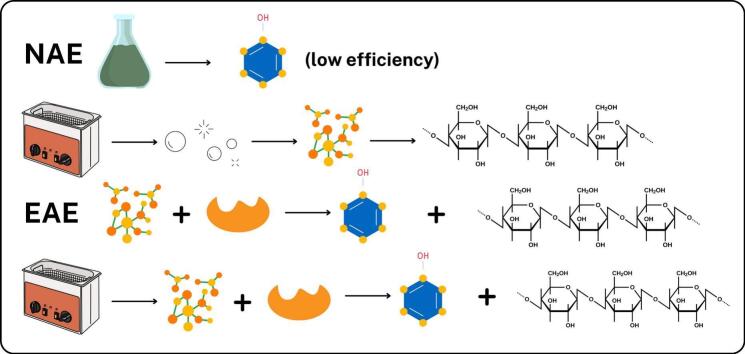

## 1 Introduction

*Elsholtzia ciliata*, commonly referred to as Vietnamese balm, is a fragrant plant belonging to the mint *Lamiaceae* family [1]. It is highly regarded for its culinary applications and therapeutic benefits in traditional medicine. This plant contains a pretty high value of bioactive constituents, including phenolic acid, flavonoids, triterpenes, and steroids. These substances are recognized for contributing a broad spectrum of therapeutic effects, such as antioxidant, anti-inflammatory, immunomodulatory, and vasodilatory [2]. Therefore, these potential characteristics highlight the necessity of extracting phenolics and flavonoids from Elsholtzia ciliata leaves.

Natural deep eutectic solvents (NADES) are viscous, transparent, uniform liquid systems that combine natural substances, including sugars, organic acids, and choline derivatives at specific molar ratios [3,4]. These solvents are formed through strong intermolecular hydrogen bonds between hydrogen bond donors (HBDs) and hydrogen bond acceptors (HBAs), resulting in eutectic mixtures with reduced melting points compared to their individual constituents [3]. NADES are well regarded for their green chemistry attributes, such as environmental safety, cost-effectiveness, biocompatibility, and tunable physicochemical properties (polarity and viscosity) [2,3]. Their biomimetic characteristics enhance the solubilization process and improve the extraction efficiency of diverse bioactive compounds. Hence, NADES represent a promising and sustainable alternative to conventional organic solvents in extraction processes [2,3]. With these advantages, numerous studies have highlighted the potential of NADES in extracting bioactive compounds from various plants and marine sources. Alexander N. Shikov et al. applied NADES to recover biological compounds from the roots of *Rhodiola rosea L* [5]. Alexander L. Semenov et al. evaluated the efficiency of NADES in extracting isoflavones from the *Pueraria lobata* roots [6]. Ekaterina D. Obluchinskaya et al. optimized the recovery of phlorotannins from the brown alga *Fucus vesiculosus* using NADES [7]. Furthermore, NADES has also been utilized to isolate bioactive compounds from *Aralia elata* var. *mandshurica by* Alyona Kaleta et al. [8], demonstrating the versatility and effectiveness of NADES in natural product extraction. The ability of acid-based NADES to co-extract elements from the roots of *Glycyrrhiza glabra* L. and associated health risks was recently studied. According to an analysis of variance (ANOVA) test, the hydrogen bond donor type plays a decisive role in the co-extraction of elements from *G. glabra* roots by NADES. A strong positive correlation between the recovery of glycyrrhizic acid and the metal pollution index (MPI) was noted. Evaluation of the MPI hazard quotient, hazard index, and chronic daily intake indicated that all tested NADES extracts of *G. glabra* roots were nontoxic and possessed no health risk for ingestion or topical application [9].

Ultrasound-assisted extraction (UAE) and enzyme-assisted extraction (EAE) are advanced and green extraction technologies widely used to isolate valuable bioactive compounds from plants. UAE transmits high-frequency sound waves through a liquid medium, inducing cavitation that disrupts plant cell structures. This physical phenomenon enhances mass transfer, accelerates solvent penetration, and improves the yield of phenolic compounds within relatively short extraction times [[10], [11], [12]]. Research has shown the UAE to be particularly effective in recovering phenolics from complex plant residues such as citrus [13], Elsholtzia ciliata [2], and pomegranate peels [10]. EAE employs hydrolytic enzymes (e.g., cellulases and pectinases) to attack the polysaccharide-rich cell walls of plant tissues. This attack loosens the structural barriers and facilitates the release of intracellular compounds under mild conditions [14]. This biochemical approach helps preserve the integrity and bioactivity of sensitive compounds while reducing reliance on traditional organic solvents. Studies have reported that combining EAE with an ultrasound synergistic method can further enhance efficiency in the extraction of phenolics from grape pomace and other agro-waste materials [15]. The mixed application of ultrasound-assisted extraction and enzyme-assisted extraction (UAE-EAE) for extracting total phenolic content (TPhC) and total flavonoid content (TFlC) from *Elsholtzia ciliata* leaves, along with the kinetic investigation of this integrated process, remains unexplored in existing literature.

Therefore, this study aims to optimize the recovery of TPhC and TFlC from *Elsholtzia ciliata* using NADES combined with UAE-EAE techniques. In addition, the extraction kinetics were investigated to find the mechanism of the process. Various NADES formulations were prepared and screened to identify a suitable solvent for maximizing extraction yield. Single-factor experiments were carried out to examine the influence of individual parameters, such as solid-to-liquid ratio (SLR), water content (WC), ultrasonic power (UP), ultrasonic temperature (UTemp), ultrasonic treatment time (UT), enzyme activity (EA), and incubation time (IT) on the extraction efficiency of TPhC and TFlC. These were followed by optimization using a Box-Behnken Design (BBD) and second-order kinetic models to investigate extraction efficiency and mechanism. The antioxidant potential of the extracts was determined through DPPH radical scavenging assays. Additionally, morphological and structural alterations in the plant material before and after the extraction process were visualized using scanning electron microscopy (SEM) and examined through X-ray diffraction (XRD) to evaluate changes at the cellular and crystallographic levels under NADES-based UAE-EAE treatment.

## 2 Materials and methods

### 2.1 Materials

The aerial parts of *Elsholtzia ciliata* (“Kinh gioi”, Vietnamese balm) were collected in Ho Chi Minh City, Vietnam (10°48′20.6″N, 106°42′08.8″E) in September 2016. The botanical identification was performed by Assoc. Prof. Dr. Hung Tran (Department of Pharmacognosy, University of Medicine and Pharmacy, Ho Chi Minh City, Vietnam). The plant nomenclature was verified according to *The Plant List* (http://www.theplantlist.org), and a voucher specimen (EC-HCMC-0916) has been deposited in the Faculty of Pharmacy, University of Medicine and Pharmacy, Ho Chi Minh City, Vietnam. Fresh Vietnamese balm (*Elsholtzia ciliata*) was procured from Kamereo Company Limited, Ho Chi Minh City, Vietnam. The leaves were manually separated from stems and subjected to drying in a laboratory hot air dryer (GW-024E, Great Win Instrument Co. Ltd., China) at 50 °C for 48 h. The dried leaves were then ground into a fine powder using a laboratory grinder (RRH-100(K), Zhejiang, China). The resulting *Elsholtzia ciliata* leaf powder (ECLP) was stored in airtight containers at room temperature to prevent moisture absorption.

### 2.2 Chemicals

Lactic acid (purity 85–90 %), 1,2-propanediol (purity 99 %), glycerol (purity ≥ 99 %), citric acid (purity 99 %), sodium phosphate dibasic heptahydrate, and sodium phosphate monobasic monohydrate were purchased from Xilong Scientific Co., Ltd., Shantou, China; Folin-Ciocalteu reagent, sodium carbonate, ethanol 96°, sodium acetate and aluminum chloride were purchased from Merck Co.Ltd, Germany; Erythritol was purchased from Smart Organic Co.Ltd., Bulgaria; Choline chloride (purity ≥ 98 %) was purchased from Biobomei Co.Ltd, China; Cellulase enzyme was purchased Brenntag Co.Ltd, Germany.

### 2.3 NADES production and screening

Natural deep eutectic solvents (NADES) were prepared by mixing a hydrogen bond donor (HBD) and a hydrogen bond acceptor (HBA) in molar ratios of HBD-to-HBA at 2:1, followed by heating and stirring at 90 °C using a magnetic stirrer (Model: MS-H380-Pro, DLAB, USA) until a transparent and homogeneous liquid was obtained with no crystallization at room temperature. The composition and ratios of the NADES used are detailed in Table 1. This study selected lactic acid and citric acid as components of NADES due to their environmental compatibility and low price. This study also aims to investigate the influence of solvent polarity and hydrogen-bonding capacity on the recovery of phenolic and flavonoid compounds. Therefore, HBAs with different numbers of hydroxyl groups were employed to combine with lactic and citric acids. A molar ratio of 2:1 was employed based on previous findings by Guang-Hui Xia et al., who utilize NADES from choline chloride and lactic acid to extract flavonoids from the rhizomes of *Polygonatum odoratum* [16].

**Table 1:** Eight NADESs for this study.

No.	Abbreviation	HBD	HBA	Molar ratio	pH	Density (g/mL)	Viscosity (cP, 10 % WC)
1	Lac − Ery	Lactic acid	Erythol	2:1	0.91	1.27 ± 0.007c	107 ± 5d
2	Lac − Cho	Lactic acid	Choline chloride	2:1	0.97	1.15 ± 0.008e	57 ± 3d
3	Lac − Pro	Lactic acid	1,2-propanediol	2:1	1.10	1.12 ± 0.009f	69 ± 4d
4	Lac − Gly	Lactic acid	Glycerol	2:1	0.60	1.19 ± 0.005d	58 ± 5d
5	Ci − Ery	Citric acid	Erythol	2:1	−1.59	1.44 ± 0.006a	1364 ± 55a
6	Ci − Cho	Citric acid	Choline chloride	2:1	−0.52	1.37 ± 0.013b	1371 ± 54a
7	Ci − Pro	Citric acid	1,2-propanediol	2:1	−1.67	1.43 ± 0.014a	653 ± 29c
8	Ci − Gly	Citric acid	Glycerol	2:1	−0.72	1.43 ± 0.012a	946 ± 34b

*HBD: hydrogen bond donor; HBA: hydrogen bond acceptor.

For extraction, 10  mL of the prepared NADES was combined with 0.5  g of Elsholtzia ciliata leaf powder (ECLP) in amber bottles with blue caps. The mixture was first subjected to ultrasound treatment in an ultrasonic bath (Model: RS30L, Rama Viet Nam Joint Stock Company, Vietnam) at 300  W for 5  min at room temperature. After ultrasonication, 0.5  mL of cellulase enzyme solution (20 U/g) was added, maintaining a sample-to-enzyme ratio of 1  g:1 mL, and the mixture was incubated at 50 °C for 30  min in a water bath (Model: Stuart SBS40/220 V/60, Cole-Parmer, England). Subsequently, 10  mL of distilled water (DI) was added to dilute the extract, which was then filtered through qualitative filter paper. The ultrasonic device has a temperature sensor that monitors the current process temperature. Ice is added to reduce the process temperature when the temperature exceeds the preset value. Conversely, when the temperature falls below the preset value, the device's heating system raises it back to the original set temperature. The filtrate was analyzed to determine the total phenolic content (TPhC) and total flavonoid content (TFlC) as indicators of extraction efficiency.

### 2.4 Single-factor experiment for UAE-EAE

Single-factor experiments were conducted to investigate the effects of SLR, WC, UP, UTemp, UT, EA, and IT on the recovery efficiency of phenolic and flavonoid compounds. In addition, these experiments were employed to define the appropriate ranges of experimental conditions for optimization studies and identify the factors exerting a significant influence on the NADES-based UAE-EAE process. ECLP was suspended in 10  mL of NADES with different SLR (1:10, 1:20, 1:30, 1:40, 1:50, and 1:60) and WC (0 to 50, with a step of 10 %). Initially, the mixture was subjected to ultrasound treatment under varying power levels (0 to 600  W, with 150  W increments), temperatures (30 °C to 70 °C, with a step of 10 °C), and durations (0, 5, 10, 15, and 20 min) to evaluate the effect of ultrasound intensity and thermal conditions on the extraction process. Then, the cellulase enzyme was introduced at different activity levels (0, 10, 20, 30, 40, and 50 U/g) to investigate the impact of enzymatic hydrolysis on the NADES-based UAE-EAE process. The enzymatic mixture was then incubated in a water bath at 50 °C for varying durations (0, 30, 60, 90, and 120 min). After the incubation period, 10  mL of DI was added to dilute the extract, and the solution was subsequently filtered to separate solid residues. The resulting filtrates were analyzed to determine TPhC and TFlC, allowing evaluation of the influence of each parameter on extraction efficiency.

### 2.5 Total phenolic content (TPhC)

The total phenolic content (TPhC) of the diluted samples was assessed through a modified Folin-Ciocalteu assay, following the approach outlined by Rodsamran et al [17]. Briefly, 250  μL of the extract was pipetted into a test tube and mixed with 250  μL of 0.2  N Folin-Ciocalteu reagent. After gentle shaking, the mixture was left to react for 5 min. Then, 500  μL of 7.5 % (w/v) sodium carbonate solution and 4000 μL of DI were added. The mixture was incubated in the dark at room temperature for 1 h. Absorbance was recorded at 765  nm using a UV–Vis spectrophotometer. TPhC was quantified using a calibration curve constructed with gallic acid and reported as milligrams of gallic acid equivalents per gram of dry weight (mg GAE/g).

### 2.6 Total flavonoid content (TFlC)

The total flavonoid content (TFlC) of the extracts was determined using a modified colorimetric assay, adapted from the methodologies described by Pham et al. and Hung & Morita [18]. In this procedure, 500  µL of the appropriately diluted extract was transferred into a test tube, followed by the sequential addition of 1000  μL of 95 % ethanol, 100  μL of 1  M potassium acetate, 100  μL of 10 % aluminum chloride, and 3300  μL of deionized water. The mixture was homogenized thoroughly and incubated at room temperature for 30 min under dark conditions. After the reaction period, the absorbance of the resulting complex was measured at 417  nm using a UV–Vis spectrophotometer. Flavonoid concentration was quantified using a quercetin calibration curve (0–200  mg/L), and results were expressed as milligrams of quercetin equivalents per gram of dry weight (mg QE/g).

### 2.7 Design experiment

#### 2.7.1 Plackett-Burman Design (PBD) model

Plackett-Burman Design (PBD) was applied to identify the most influential variables affecting the extraction efficiency of TPhC and TFlC from ECLP. Based on preliminary screening results in the UAE-EAE section, two optimized NADES formulations, each yielding the highest TPC and TFC, respectively, were selected as solvents for the model evaluation. Seven independent variables were examined at two levels (“-1″ for the lower and ”+1″ for the higher, with “0″ representing the baseline condition identified from single-factor trials). These variables included solid-to-liquid ratio (SLR), water content in the solvent (WC), ultrasonic power (UP), ultrasonic temperature (UTemp), ultrasonic time (UT), enzyme activity (EA), and incubation time (IT). A total of 12 experimental runs were designed, and the relationship between factors and responses is represented in equation (1):

(1) $$F=\sum_{i=1}^{n}\alpha_{i}P_{i}$$

Where F represent the predicted response value, in other words, dependent factors F_1_ and F_2_ were the TPhC (mg GAE/g) and TFlC (mg QE/g), respectively; P_1_ through P_7_ correspond to the seven tested variables; α_i_ denotes the regression coefficient of the i^th^ factor; and n is the number of examined factors (n = 7). Factors with p-values less than 0.05 were considered to have a statistically significant impact on the phenolic and flavonoid yields from ECLP.

#### 2.7.2 Box-Behnken Design (BBD) model

After identifying significant variables from the Plackett-Burman Design, the extraction process for ECLP was further optimized using a Box-Behnken Design (BBD). Seven independent variables selected from the preliminary screening: SLR, WC, UP, UTemp, UT, EA, and IT were investigated at three coded levels (−1, 0, +1), as presented in Table 2. A total of 42 experimental runs for TPhC and 14 experimental runs for TFlC, each including two center points, were conducted to explore the influence of selected factors on the extraction outcomes. The relationship between the response values and the experimental factors was modelled using a second-order polynomial equation using equation (2):

(2) $$F=I_{0}+\sum_{i=1}^{m}I_{i}P_{i}+\sum_{i=1}^{m}I_{\mathrm{ii}}P_{i}^{2}+\sum_{i=1}^{m}\sum_{j=1}^{m}I_{\mathrm{ij}}P_{i}P_{j}$$

Where F represents the predicted response value (TPC or TFC, expressed in mg GAE/g or mg QE/g), I_0_ is the intercept, I_i_, I_ii_, and I_ij_ are the coefficients for linear, quadratic, and interaction terms, respectively. P_i_ and P_j_ denote the coded levels of the independent variables, and *m* is the number of investigated factors (m = 5 for TPhC and m = 3 for TFlC measurement). To assess the model's accuracy, the prediction error (%) was calculated using the following formula (equation (3)):

(3) $$\mathrm{Predictionerrors}=\frac{\left|themeanofmeasuredvalue-predictedvalues\right|}{\mathrm{themeanofmeasuresvalue}}\times 100$$

**Table 2:** Experimental design factors and their levels for optimizing the UAE-EAE.

Variables	Independent factors	Units	The value of independent factors		
			Lower (−1)	Proper (0)	Higher (+1)
TPhC measurement					
P_1_	SLR	g/mL	1:40	1:50	1:60
P_4_	UTemp	^o^C	40	50	60
P_5_	UT	min	5	10	15
P_6_	EA	U/g	0	10	20
P_7_	IT	min	0	30	60
TFlC measurement					
P_2_	WC	%	0	10	20
P_3_	UP	W	150	300	450
P_5_	UT	min	5	10	15

* TPhC: Total phenolic content; TFlC: Total flavonoid content**;** SLR: Solid-to-liquid ratio; WC: Water content; UP: Ultrasonic power; UTemp: Ultrasonic temperature; UT: Ultrasonic time; EA: Enzyme activity; IT: Incubation time.

### 2.8 Second-order extraction kinetic models

The extraction kinetics of phenolic and flavonoid compounds during the UAE-EAE process were investigated by applying a second-order kinetic model. This model, initially developed by Ho and McKay [19], describes the rate at which bioactive compounds are transferred from the plant matrix into the solvent phase:

(4) $$\frac{dc_{t}}{\mathrm{dt}}=k{\left(c_{\mathrm{eq}}-c_{t}\right)}^{2}$$

Where: c_t_ is the concentration of phenolics or flavonoids at any time (mg/g), c_eq_ is the concentration of phenolics or flavonoids at the equilibrium point (mg/g), k: the second-order extraction rate constant (g/mg.min), and t is the extraction time (min)

By integrating this differential equation (4) and rearranging the terms, a linearized form is obtained:

(5) $$\frac{t}{c_{t}}=\frac{t}{c_{\mathrm{eq}}}+\frac{1}{kc_{\mathrm{eq}}^{2}}$$

Which follows the structure of a linear equation y=at+b, where:$y=\frac{t}{c_{t}};a=\frac{t}{c_{\mathrm{eq}}};andb=\frac{1}{kc_{\mathrm{eq}}^{2}}$

The initial extraction rate h (mg/min·g) can be calculated as:

(6) $$h=kc_{\mathrm{eq}}^{2}$$

By plotting $\frac{t}{c_{t}}$ versus t using equation (5), the slope and intercept of the fitted line allow for estimation of both k and $C_{\mathrm{eq}}$. Furthermore, the temperature dependence of the extraction process was evaluated using the Arrhenius equation (7):

(7) $$k=A∙e^{-\frac{\mathrm{Ea}}{\mathrm{RT}}}$$

### 2.9 Antioxidant activity determination

The antioxidant properties of Elsholtzia ciliata leaf extracts were evaluated using three established assays: DPPH (2,2-diphenyl-1-picrylhydrazyl), ABTS (2,2′-azino-bis(3-ethylbenzothiazoline-6-sulfonic acid)), and FRAP (ferric ion reducing antioxidant power). The DPPH and ABTS methods followed the methodologies of Rodsamran and Sothornvit [20], while the FRAP assay was adapted from Müller et al. [21] with slight changes to fit the experimental setup.

#### 2.9.1 DPPH assay

For the DPPH assay, 500  μL of the extract was mixed with 3500  μL of a 100  μM DPPH solution prepared in absolute ethanol. The reaction mixture was shielded from light and left for 30 min to allow the reaction to proceed. Absolute ethanol served as the blank, and absorbance was recorded at 515  nm. A Trolox calibration curve (0–40  μM) was applied to quantify the antioxidant capacity.

#### 2.9.2 ABTS assay

In the ABTS test, 100 µL of the extract was combined with 3900 μL of ABTS•^+^ solution and left in the dark for 30 min. The ABTS•^+^ radical was generated by reacting equal parts of 7.4 mM ABTS and 2.45 mM potassium persulfate solutions in the dark until the absorbance reached 1.00 at 735 nm. Blank of this assay is DI. The absorbance of the test samples was measured at 735 nm, and antioxidant activity was estimated based on a Trolox calibration curve between 0 and 600 μM. Results from both methods were reported in micromoles of Trolox equivalents per gram of dry weight (μM TE/g).

#### 2.9.3 FRAP assay

In the FRAP assay, a mixture of 3900  μL FRAP reagent and 100  μL extract was incubated for 30 min at room temperature under light-protected conditions. The reagent was prepared by combining acetate buffer (pH 3.6, 80  mL), FeCl_3_·6H_2_O (8 mL, 20  mM), and TPTZ (2,4,6-tripyridyl-s-triazine, 8 mL, 10  mM) in HCl solution (40  mM). Absorbance was recorded at 593  nm using a spectrophotometer, and antioxidant capacity was expressed as milligrams of Trolox equivalents per gram of extract (mg TE/g).

### 2.10 Surface morphology and X-ray diffraction (XRD) of ECLP

The morphological characteristics of *Elsholtzia ciliata* leaf powder (ECLP) before and after extraction treatment were observed using SEM (Model: Hitachi S-4800). Sample preparation was carried out according to the procedure adapted from Patil et al. [22]. The powder samples were mounted onto a carbon adhesive tape on a metal stub to ensure sample stability during analysis. Afterwards, the specimens were coated with a thin layer of gold using a vacuum sputter coater to enhance conductivity. Surface structure changes were then examined under varying magnifications using SEM.

To evaluate structural modifications in crystallinity, treated and untreated ECLP samples were analyzed via XRD using a D8 Advance diffractometer (Bruker, Germany), following the method outlined by Agarwal et al. [19]. The crystallinity index (CCI) of cellulose was determined based on the relative intensity of crystalline and amorphous regions in the diffraction pattern, calculated using the following equation (8):

(8) $$\mathrm{CCI}=\frac{I_{\mathrm{crys}}-I_{\mathrm{amp}}}{I_{\mathrm{crys}}}\times 100\%$$

Where I_crys_ and I_amp_ represent the intensities of the crystalline and amorphous peaks at approximately 22.5° and 18° (2θ), respectively.

### 2.11 Statistical analysis

All experimental data were presented as mean values accompanied by standard deviation (±SD), based on three independent replicates for each condition. Statistical evaluations were performed using Minitab 19 (Minitab Inc., USA) and Design-Expert 13 (Stat-Ease Inc., USA), applying analysis of variance (ANOVA) at a 95 % confidence level to determine the significance of the results. Graphs and visual data interpretations were generated using OriginPro 2024 (OriginLab Corporation, USA).

## 3 Results and discussion

### 3.1 NADES characteristics

#### 3.1.1 pH

Table 1 presents the pH values of NADES. The pH values of NADESs ranged from −1.67 to 1.10. The Lac-Pro solvent showed the highest pH, while Ci-Pro displayed the lowest. The particularly low pH of citric acid-based NADESs is consistent with the firm acidity of citric acid, which has a low first dissociation constant (pKa_1_ ≈ 3.1) [23]. This observation aligns with previous findings by Benvenutti et al. [24], who also reported citric acid-based NADESs as having the lowest pH among various acid-based formulations.

The pH differences among the NADES types were also influenced by the choice of hydrogen bond acceptor (HBA). The pH trend in lactic acid-based systems: Lac-Pro > Lac-Cho > Lac-Ery > Lac-Gly. This trend suggests that the basicity and hydrogen-bonding interactions of HBAs such as 1,2-propanediol and choline chloride may help suppress proton dissociation. A similar trend was observed in citric acid-based systems, where Ci-Cho and Ci-Gly exhibited slightly higher pH values than Ci-Pro and Ci-Ery. There was no correlation between the pH of NADES and the extraction efficiency of bioactive compounds in this study (Fig. S1). This finding contradicts previous studies reporting enhanced anthocyanin recovery at lower pH. This difference can be attributed to the chemical stability of different compounds. Anthocyanins are more stable under strongly acidic conditions, thus, exhibit higher recovery at lower pH [25]. Phenolic compounds remain stable within a narrow pH range of 1–2. The capture of phenolics by citric acid-based NADES can be lower than that of lactic acid-based NADES [26]. Additionally, except for Lac-Cho, citric acid-based solvents achieved higher recovery yields of phenolics than lactic acid-based solvents. It can be ascribed to the greater extraction yield of the anthocyanin fraction.

#### 3.1.2 Density

The density of NADES is demonstrated in Table 1. The measured densities of the eight NADES varied between 1.12  g/mL for Lac–Pro and 1.44 g/mL for Ci–Pro (Table 1). As a key physicochemical parameter, solvent density plays a critical role in the process design and modelling of mass transfer dynamics, influencing phase separation efficiency and flow behavior [27,28]. Moreover, NADES exhibit densities exceeding those of water and common organic solvents like ethanol, methanol, and hexane. This attribute facilitates uniform phase distribution and improved recovery in liquid–liquid separation operations.

The nature of the HBA and HBD components governs density in NADES formulations. Organic acids with longer alkyl chains or multiple hydroxyl groups yield denser eutectic liquids due to increased intermolecular hydrogen bonding and tighter molecular packing. The density of citric-acid-based NADES is higher than that of lactic-acid-based ones, with 1.44 g/mL of Ci-Pro compared to 1.12 g/mL of Lac-Pro, owing to citric acid's greater number of –OH functionalities. Moreover, the molecular arrangement shifts with the composition of the NADES, where organic-acid-based NADES tend to be denser than sugar and polyol-based NADES. This trend is attributed to denser hydrogen bonding networks and organic acids' comparatively compact molecular structure versus larger sugar molecules [27,29].

#### 3.1.3 Viscosity

The viscosities of the eight investigated NADES varied substantially, ranging from 57 cP to 1371 cP (Table 1). The Lac-Cho (57 cP) and Lac-Gly (58 cP) solvents demonstrated the lowest viscosity, while Ci-Ery (1364 cP) and Ci-Cho (1371 cP) solvents showcased the highest. Viscosity constitutes a pivotal physicochemical parameter of NADES, as it has a direct influence on extraction performance [30]. Citric-based NADES exhibit high viscosities (>100 cP) at room temperature. The elevated viscosity of citric acid-based NADES arises from the multifunctional character of citric acid, which possesses hydroxyl and three carboxyl groups capable of establishing extensive hydrogen-bonding networks with hydrogen bond donors [31]. The exceedingly high viscosities recorded for Ci-Cho and Ci-Ery are indicative of the formation of highly ordered supramolecular assemblies with polyhydroxylated donors. Such dense hydrogen-bond networks substantially limit fluidity, hinder solute mass transfer, and pose challenges for their practical application in extraction systems [30]. Lac-Cho exhibited one of the lowest viscosities (57 cP). Lactic acid bears only a single hydroxyl and a single carboxyl group. This structure results in forming a moderate number of hydrogen-bonding interactions with polyols, which account for the relatively reduced viscosities observed in lactic acid-based NADES.

#### 3.1.4 Characterizing the functional groups of NADES

FTIR spectra of the eight NADES formulations (Fig. S2) confirm the presence of key functional groups and their intermolecular interactions. The broad O-H stretching band between 3510–3340  cm^–1^ signifies strong hydrogen-bond networks formed between the hydroxyl groups of polyols (like glycerol, erythritol, and 1,2-propanediol) and carboxylic acids (lactic or citric acid), as well as between the hydroxyls and the N-H groups in choline chloride-based systems. This observation aligns with previous findings in choline chloride-lactic acid NADES by O. N. Pozharitskaya et al. [32], where similar bands indicated O-H···Cl and O-H···COOH interactions.

A sharp C=O stretching peak at 1730–1709  cm^–1^ appears in all spectra, reflecting the carboxyl moieties from both acids. Citric acid-based NADES (Ci-Cho and Ci-Pro) exhibit stronger C=O signals, likely due to multiple carboxyl groups, supporting denser hydrogen bonding. A minor band at 1645  cm^–1^ is attributed to C=C stretching. In the fingerprint region (1200–900  cm^−1^), overlapping signals consistent with C-O, C-C, and O-C-O stretching and bending modes appeared. Choline chloride-based NADESs exhibited distinct peaks at 1480  cm^−1^ (–CH_3_ bending) and 1415  cm^−1^ (–CH scissoring), highlighting additional contributions from the tertiary ammonium group. These features' complexity and relative intensity underscore the denser hydrogen-bond network fostered by choline chloride inclusion. These spectral signatures, broad O-H/N-H absorption, pronounced C=O stretching, and fingerprint region complexities uniformly confirm the formation of stable NADES via extensive hydrogen-bonding between polyols or choline chloride and lactic or citric acid components.

### 3.2 NADES screening and method selection

**Fig. 1:**
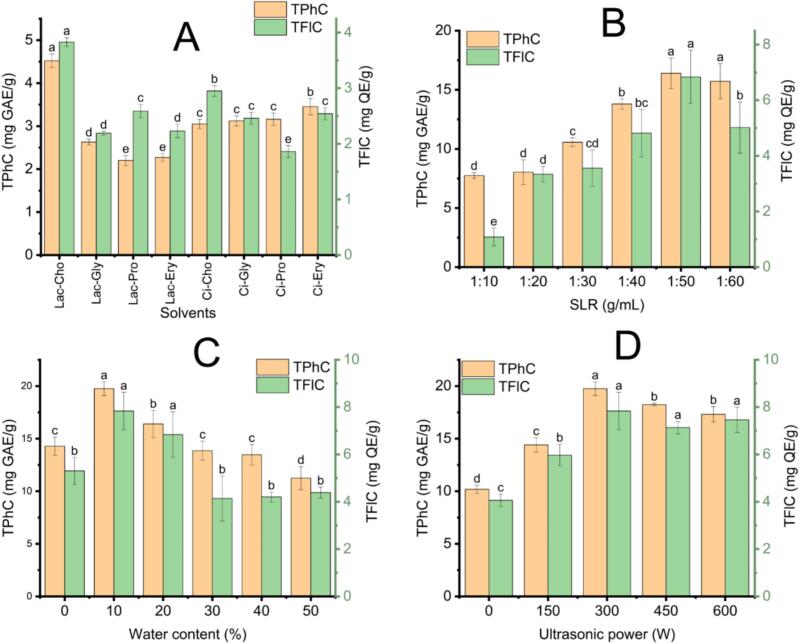
Influence of solvent types and extraction parameters on the recovery of phenolics and flavonoids using Lac-Cho in the UAE-EAE process; (A): The effect of solvent systems on TPhC and TFlC; (B): The effect of solid-to-liquid ratios on TPhC and TFlC; (C): The effect of water content on TPhC and TFlC; (D): The effect of applied ultrasonic power on TPhC and TFlC. Distinct letters (a, b, c, d, e) denote statistically significant differences among treatment groups. TPhC: Total phenolic content; TFlC: Total flavonoid content.

A total of eight NADESs were formulated using two hydrogen bond donors (HBDs: lactic acid and citric acid) in combination with four hydrogen bond acceptors (HBAs: erythritol, choline chloride, 1,2-propanediol, and glycerol) to evaluate their efficiency in extracting phenolic and flavonoid compounds from ECLP. These systems were tested under fixed extraction conditions, including an SLR of 1:20 (g/mL), UP of 300  W, UTemp of 50 °C, UT of 5 mins, EA of 20 U/g, IT of 30 mins, and incubation temperature of 50 °C. As presented in Fig. 1A, Lac-Cho achieved the highest extraction yields for TPhC (4.52 ± 0.16  mg GAE/g) and TFlC (3.83 ± 0.08  mg QE/g). In contrast, Lac-Pro got the lowest extraction yield for TPhC (2.20 ± 0.12  mg GAE/g), and Ci-Pro had the lowest yield of TFlC (1.86 ± 0.11  mg QE/g).

Polarity plays a pivotal role in the extraction efficiency of NADES, as it governs the solubility of bioactive compounds like phenolics and flavonoids. When the polarity of the solvent closely matches that of the solute, solubility is enhanced, leading to better mass transfer and higher extraction yields [33]. The partition coefficient (log P) is commonly used to estimate polarity: lower log P values correspond to more polar compounds and solvents. Among the hydrogen bond donors (HBDs), lactic acid has a relatively low log P value of 0.72 [34], indicating high polarity. Similarly, among the hydrogen bond acceptors (HBAs), choline chloride has been shown to exhibit strong ionic and polar character, promoting better interaction with hydrophilic solutes like phenolics. Additionally, choline chloride significantly lowers viscosity and improves mass transfer during UAE-EAE [35]. Regarding Lac-Pro and Ci-Pro, the combination of polar acids with less polar HBA (1,2-propanediol) showed poorer performance due to weaker polarity matching with target analytes. In addition to polarity, the physicochemical properties of NADES impact their ability to recover bioactive compounds. Solvents with extreme acidity may lead to degradation and ionization of sensitive compounds, reducing extraction yield [36,37]. In this study, Lac-Cho (pH ≈ 0.97) provided a suitable environment for preserving and dissolving phenolic compounds. Strongly acidic NADESs like Ci-Pro (pH ≈ −1.67) and Ci-Ery (pH ≈ −1.59) may destabilize polyphenolic structures. Furthermore, solvent density influences phase dispersion and mass transfer efficiency. High-density NADESs like Ci-Pro (1.44  g/mL) may hinder solvent penetration into the plant matrix. Lac-Cho (1.16  g/mL) maintained a more favorable balance, facilitating solute diffusion [32]. Therefore, Lac-Cho was selected as the solvent for phenolic and flavonoid extraction for subsequent experiments.

### 3.3 Single factor experiments

#### 3.3.1 Effect of solid: Liquid ratios

The impact of the solid-to-liquid ratio (SLR) using Lac-Cho NADES on the extraction efficiency of phenolic and flavonoid compounds from ECLP was investigated under fixed extraction conditions: WC of 20 %, UP of 300 W, UTemp of 50 °C, UT of 5  min, EA of 20 U/g, IT of 30 min, and incubation temperature of 50 °C. As shown in Fig. 1B, SLR values ranging from 1:10 to 1:60 were assessed. At the lowest ratio of 1:10 g/mL, TPhC and TFlC exhibited the minimum recorded values, with 7.74 ± 0.26  mg GAE/g and 1.08 ± 0.32  mg QE/g, respectively. This limited yield is attributed to insufficient solvent volume, which restricts the solubilization and diffusion of bioactive compounds into the liquid phase. High viscosity and limited solvent-solid interactions under these conditions can inhibit efficient mass transfer from plant cell interiors to the surrounding medium [38]. As the SLR increased, a marked improvement in extraction efficiency was observed. At the ratio of 1:50 g/mL, TPC and TFC values peaked at 16.4 ± 1.29  mg GAE/g and 6.83 ± 0.96  mg QE/g, representing approximately 2.12-fold and 6.32-fold increases, respectively, compared to the ratio of 1:10 g/mL. This enhancement is due to the greater concentration gradient of solutes between the plant matrix and solvent, as well as improved solute–solvent contact. These factors promote solute diffusion and accelerate the dissolution of target compounds [39,40]. However, further increasing the SLR to 1:60 g/mL did not yield additional improvement. Slight decreases in TPC and TFC were noted, possibly due to excessive solvent volume leading to lower extraction driving force, solubilization of impurities, and matrix degradation [38]. Similar findings were reported by C. Liu et al. [41], who documented that increasing SLR enhanced flavonoid recovery from lotus leaves in a UAE-EAE setup. Therefore, SLR 1:50 g/mL was determined as the appropriate condition for maximizing the recovery of phenolics and flavonoids in subsequent experimental optimization.

#### 3.3.2 Effect of water content in NADES

The impact of water content on the extraction efficiency of phenolics and flavonoids was evaluated under constant experimental conditions: SLR of 1:50  g/mL, UP of 300 W, UTemp of 50 °C, UT of 5  min, EA of 20 U/g, IT of 30 min, and incubation temperature of 50 °C. As illustrated in Fig. 1C, the highest recovery of TPhC (19.75 ± 0.65  mg GAE/g) and TFlC (7.84 ± 0.79  mg QE/g) was achieved when ten percent of water was incorporated into the NADES. This enhancement is due to the reduced viscosity caused by moderate water addition, which facilitates mass transfer and improves diffusion rates. It also allows better solvent penetration into plant tissue without disrupting the internal hydrogen bonding of the NADES system [42,43]. However, when the water content exceeded 10 %, a progressive decline in both TPhC and TFlC was observed. This drop is caused by excessive water disrupting the supramolecular structure of NADES and weakening the hydrogen bonds between its components. The eutectic mixture is effectively transformed into a diluted aqueous system (as illustrated in Fig. S3). As a result, the ability of the solvent to solubilize and interact with bioactive compounds diminishes significantly [19]. These findings align with prior research by Shang et al. [44], which also demonstrated reduced flavonoid yields at high water content in NADES-based extraction systems. Olga N. Pozharitskaya et al. investigated the physicochemical and antimicrobial properties of lactic acid-based NADESs with varying water contents. The study found that increasing the water fraction reduced viscosity and surface tension, with viscosity decreasing by up to 50 % upon the addition of 10 % water and surface tension dropping by 46.7 % in the lactic acid-based NADES [32]. In theory, these reductions should facilitate mass transfer and improve the recovery of phenolic and flavonoid compounds. However, our study demonstrated the opposite trend, as higher water content decreased the recovery of phenolics and flavonoids. The disruption of the hydrogen-bonding network diminishes the solvent's ability to interact with these compounds. These findings suggest that the number of hydrogen bonds plays a more decisive role in extraction processes than viscosity or surface tension. Furthermore, Olga N. Pozharitskaya et al. showed that the antimicrobial activity of NADESs declined by approximately two-fold to eight-fold upon the addition of 40 % water [32]. This reduction was linked to increased water activity, which weakened the inhibitory effect on microbial growth. Therefore, ten percent of water content in Lac-Cho was identified as the suitable condition for maximizing the extraction of phenolic and flavonoid compounds from ECLP.

#### 3.3.3 Effect of ultrasonic power

The influence of ultrasonic power (UP) ranging from 0 to 600 W on TPhC and TFlC was investigated under controlled parameters, including SLR of 1:50  g/mL, WC of 20 %, UTemp of 50 °C, UT of 5 min, EA of 20 U/g, IT of 30 min, and incubation temperature of 50 °C. As illustrated in Fig. 1D, the results revealed that TPhC and TFlC yield the highest at 19.75 mg GAE/g and 7.84 mg QE/g, respectively, when an ultrasonic power of 300 W was applied. Ultrasonic cavitation generates large bubbles that collapse violently, producing microjets and intense shear forces. These effects enhance solvent penetration and cell wall disruption, promoting efficient mass transfer and compound release [45]. However, further increasing the UP to 600 W led to a 12 % decline in TPhC and a 5 % reduction in TFlC. This drop in efficiency may be attributed to thermal degradation of sensitive bioactive molecules and reduced cavitation activity at excessive power levels [46]. These results are consistent with findings from Hiranpradith et al. [46], who reported a similar trend in phenolic extraction from *Commiphora gileadensis* leaves under varying ultrasonic intensities. Hence, 300 W was selected as the appropriate UP for further extraction optimization of phenolic and flavonoid compounds.

#### 3.3.4 Effect of ultrasonic temperature

**Fig. 2:**
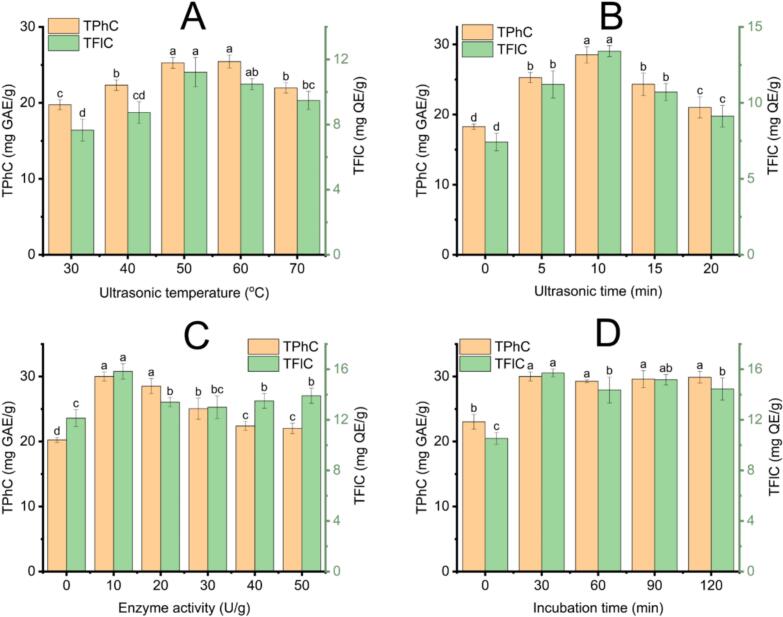
Influence of extraction parameters on the recovery of phenolics and flavonoids using Lac-Cho in the UAE-EAE process; (A): The effect of ultrasonic temperature on TPhC and TFlC; (B): The effect of ultrasonic time on TPhC and TFlC; (C): The effect of enzyme activity on TPhC and TFlC; (D): The effect of incubation time on TPhC and TFlC. Distinct letters (a, b, c, d) denote statistically significant differences among treatment groups. TPhC: Total phenolic content; TFlC: Total flavonoid content.

The impact of ultrasonic temperature (UTemp) on the recovery efficiency of TPhC and TFlC was assessed over a range of 30 °C to 70 °C, with all other extraction conditions held constant SLR of 1:50  g/mL, WC of 20 %, UP of 300 W, UT of 5 min, EA of 20 U/g, IT of 30 min and incubation temperature of 50 °C. The results are presented in Fig. 2A. An increase in temperature from 30 °C to 60 °C led to a significant enhancement in extraction yield. The highest TPhC was recorded at 60 °C (25.43 ± 0.86  mg GAE/g), while TFlC peaked at 50 °C (11.22 ± 0.90  mg QE/g). This improvement results from increased molecular kinetic energy at higher temperatures, which lowers NADES viscosity. As a consequence, molecular mobility is enhanced, facilitating diffusion into plant tissues and thereby promoting a more efficient release of solutes [47,48]. However, further increasing the temperature beyond 60 °C did not result in additional yield improvement. This trend could be due to reduced surface tension at higher temperatures, which weakens the cavitation intensity during ultrasonication. Diminished cavitation causes less efficient bubble collapse and reduced solvent penetration, leading to hindering mass transfer and the release of bioactive compounds [49]. Al-Dhabi et al. [39] observed a similar trend in extraction efficiency during phenolic extraction from coffee grounds. Based on these findings, a UTemp of 50 °C was chosen as the compromise temperature for the UAE-EAE process.

#### 3.3.5 Effect of ultrasonic time

The influence of ultrasonic time (UT) on the extraction performance of TPhC and TFlC was systematically investigated under consistent experimental conditions, including SLR of 1:50  g/mL, WC of 20 %, UC of 300 W, UTemp of 50 °C, EA of 20 U/g, IT of 30 min, and incubation temperature of 50 °C. As illustrated in Fig. 2B, extending the UT from 0 to 10 min led to a notable enhancement in both TPhC and TFlC yields. This trend is due to intensified cavitation, which improves solvent penetration and breaks down plant cell walls. The release of intracellular phenolic and flavonoid compounds into the solvent is accelerated [48]. Maximum yields were achieved at 10 min of ultrasonication, with TPhC reaching 28.52 ± 1.16  mg GAE/g and TFlC attaining 13.4 ± 0.36  mg QE/g. However, extending the UT beyond this point had a detrimental effect. When increased to 20 min, TPhC and TFlC significantly declined, dropping to 21.02 ± 1.51  mg GAE/g and 9.12 ± 0.72  mg QE/g, respectively. This can be explained by the heating effect and prolonged time to ultrasound treatment, which leads to structural breakdown and decreased extraction efficiency [50]. A similar trend was observed in the study by Pan et al. [51], which indicates reduced flavonoid yields from hawthorn seeds after extended ultrasonic treatment. Thus, a UT of 10 min was chosen as a proper duration for achieving high recovery of bioactive compounds from ECLP.

#### 3.3.6 Effect of enzyme activity

Enzyme activity (EA) is a critical variable that significantly affects the extraction efficiency of TPhC and TFlC. This experiment assessed EA in the 0–50 U/g range under constant extraction conditions: SLR of 1:50  g/mL, WC of 20 %, UC of 300 W, UTemp of 50 °C, UT of 10 min, IT of 30 min, and incubation temperature of 50 °C. The results are presented in Fig. 2C. An EA of 10 U/g yielded the highest levels of TPhC (30.00 ± 0.70  mg GAE/g) and TFlC (15.83 ± 0.61  mg QE/g). This yield marks a significant improvement compared to the absence of enzyme (20.24 ± 0.39  mg GAE/g, 12.13 ± 0.67 mg QE/g, respectively). The formation of enzyme–substrate complexes is intensified, thereby promoting the hydrolysis of plant cell wall components such as cellulose and hemicellulose. This process facilitates the release of phenolics and flavonoids into the solvent [52]. However, further increasing EA to 50 U/g resulted in noticeable declines in both TPhC and TFlC by factors of approximately 1.36 and 1.14, respectively. High enzyme concentrations may break hydrogen bonding networks and increase the release of free phenolics into the solvent. These compounds may migrate toward the solvent interface at elevated temperatures and long reaction times. They become more susceptible to oxidation or environmental degradation [53]. Similar behavior was also observed in the work of Islam et al. [54], who reported a decline in polyphenol yield from banana peels when enzyme levels exceeded optimal thresholds. These findings confirm that 10 U/g is the effective enzyme dosage for the extraction of TPhC and TFlC from *Elsholtzia ciliata* leaf powder (ECLP).

#### 3.3.7 Effect of enzyme incubation time

The influence of enzyme incubation time (IT) on the extraction of TPhC and TFlC from ECLP using Lac-Cho as the solvent was evaluated under fixed conditions: SLR of 1:50  g/mL, WC of 20 %, UC of 300 W, UTemp of 50 °C, UT of 10 min, EA of 10 U/g, and incubation temperature of 50 °C. The results are depicted in Fig. 2D. At zero minutes of incubation, TPhC and TFlC extraction yields were minimal. The absence of pre-incubation prevents enzymatic interaction with the plant cell walls, thereby inhibiting hydrolysis of cellulose structures and restricting the release of embedded bioactive [53]. As IT increased, yields improved significantly, peaking at 30 min with 30.00 ± 0.70  mg GAE/g for TPhC and 15.71 ± 0.31  mg QE/g for TFlC. Cellulase activity breaks down cellulose chains, forming surface fissures and pores in the plant matrix. The pore formation enhances solvent penetration and improves compound release [55]. However, when incubation time was extended to 120 min, TPhC and TFlC declined slightly to 29.87 ± 0.89  mg GAE/g and 14.44 ± 0.88  mg QE/g, respectively. Prolonged exposure may lead to structural degradation or oxidation of the released compounds, particularly at the solvent interface, thus reducing the overall recovery efficiency [56]. A similar trend has been observed in studies of R. Liu et al. [57], who reported flavonoid extraction from *Acanthopanax senticosus*. A 30-minute enzyme incubation was the most effective duration for the recovery of phenolic and flavonoid compounds from ECLP.

### 3.4 Evaluating the degree of conditional effects on the recovery of bioactive compounds

The PBD model was constructed to evaluate the influence of seven independent variables on TPhC and TFlC extraction yields. The variables included SLR (P_1_), WC (P_2_), UP (P_3_), UTemp (P_4_), UT (P_5_), EA (P_6_), and IT (P_7_). Experimental results and the corresponding ANOVA are presented in Table S1 and Table S2, respectively. Additionally, the key statistical metrics of the model, including the coefficient of determination (R^2^), the adjusted coefficient of determination (Adjusted R^2^), and the predicted coefficient of determination (Predicted R^2^), were calculated. The mathematical relationships describing the correlation between extraction efficiency and the experimental factors are provided in equations (9), (10).

(9) $$Y_{\mathrm{TPhC}}=20.354-2.961P_{1}-0.762P_{2}+1.373P_{4}-0.972P_{5}+2.656P_{7}$$

(10) $$Y_{\mathrm{TFlC}}=9.01-2.21P_{1}+0.503P_{2}-0.524P_{6}$$

**Fig. 3:**
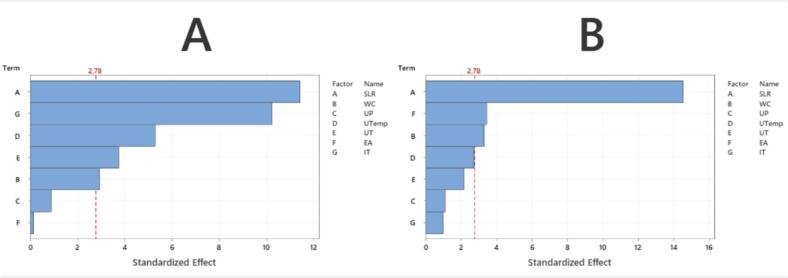
Pareto diagrams produced from the Plackett-Burman Design for TPhC (A) and TFlC (B). SLR: Solid-to-liquid ratio; WC: Water content; UP: Ultrasonic power; UTemp: Ultrasonic temperature; UT: Ultrasonic time; EA: Enzyme activity; IT: Incubation time.

In the PBD model, variables with absolute t-values exceeding 2.78 or p-values less than 0.05 were considered statistically significant, while those below this threshold were considered non-significant. As illustrated in Fig. 3A, the SLR was identified as the most influential factor affecting phenolic recovery from ECLP. This observation is consistent with findings by Blamo Jr et al. [58]. This research reported that the liquid-to-solid ratio was the most influential factor affecting the recovery efficiency of phenolic compounds from the red alga *Laurencia* intermedia using ultrasound-assisted extraction. Other factors that decreased the order of impact included IT, UTemp, UT, and WC. As shown in Fig. 3B, SLR, EA, and WC were identified as the predominant variables affecting flavonoid extraction efficiency. To summarize, the optimization of TPhC recovery via the UAE-EAE method was influenced by SLR, WC, UTemp, UT, and IT. In contrast, the key parameters affecting flavonoid extraction were identified as SLR, WC, and EA.

### 3.5 Optimization of the UAE-EAE process

#### 3.5.1 Regression models

Five significant variables influencing TPhC, namely SLR (P_1_), WC (P_2_), UTemp (P_4_), UT (P_5_), IT (P_7_); and those affecting TFlC, including SLR (P_1_), WC (P_2_), EA (P_6_), were subjected to the optimization of the UAE-EAE process using the BBD model. The outcomes of 42 experimental trials for TPhC and 14 trials for TFlC are summarized in Table S3. The estimated regression coefficients of the quadratic polynomial models, along with the results of ANOVA and model significance levels, are presented in Table S4. For the TPhC model, the polynomial regression was highly significant (p < 0.0001), indicating a strong fit. The determination coefficients R^2^ = 96.80 % and adjusted R^2^ = 93.75 % confirmed the model's reliability in predicting phenolic yield within the defined parameter space. Similarly, for the TFlC model, a strong agreement between the actual and predicted data was observed, as evidenced by the high coefficients of determination R^2^ = 97.61 % and adjusted R^2^ = 92.24 %. These results indicate that the developed models are statistically robust and can be reliably applied to predict TPhC and TFlC. The fitted responses are represented by the second-order polynomial equations (11), (12).

(11) $$Y_{\mathrm{TPhC}}=34.45+4.79P_{1}-1.50P_{2}+0.77P_{4}-1.24P_{5}-4.37P_{1}P_{7}+3.33P_{1}P_{4}-1.40P_{5}P_{7}-4.83P_{1}^{2}-6.53P_{2}^{2}-5.04P_{4}^{2}-7.04P_{5}^{2}-5.70P_{7}^{2}$$

(12) $$Y_{\mathrm{TFlC}}=14.86-2.20P_{2}-2.61P_{6}^{2}$$

TPhC was not significantly influenced by these variables: UP, EA, and IT (p > 0.05). ANOVA analysis revealed that the linear effects of P_1_, P_2_, P_4_, P_5_, the interaction terms (P_1_P_7_, P_1_P_4_, P_5_P_7_), and the quadratic effects (P_1_^2^, P_2_^2^, P_4_^2^, P_5_^2^, P_7_^2^) were statistically significant. While SLR and UTemp had a positive linear impact on TPhC, their quadratic effect was negative. The combination of SLR and UTemp positively affected TPhC. The interactions of SLR and IT, as well as UT and IT, negatively influenced TPhC.

**Fig. 4:**
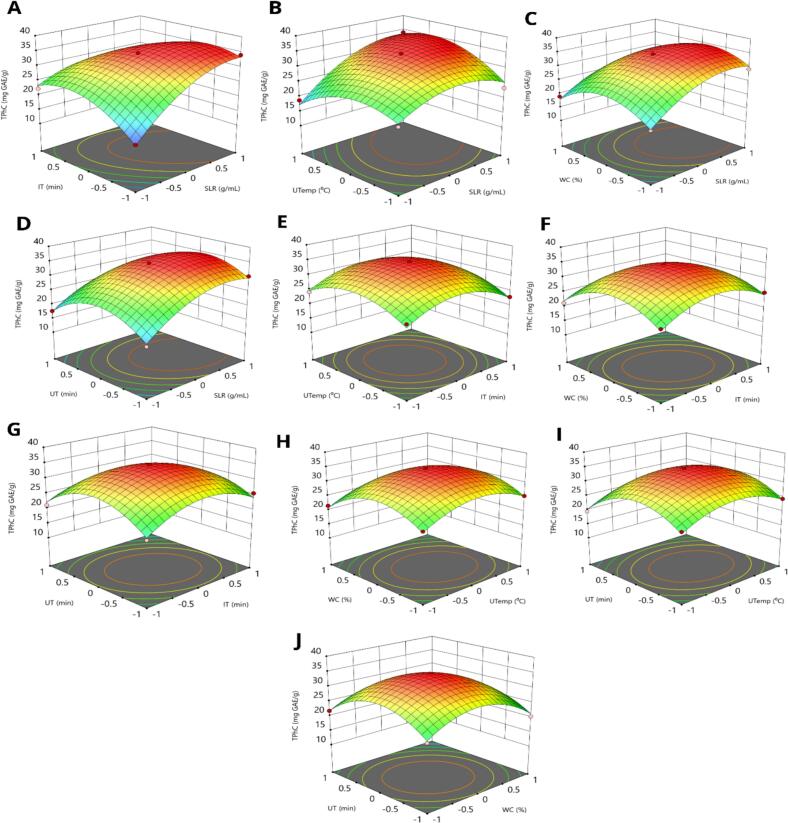
Three-dimensional response surface plots illustrating the interactive effects of UAE-EAE process on TPhC; (A): incubation time and solid-to-liquid ratios; (B): ultrasonic temperature and solid-to-liquid ratios; (C): water content and solid-to-liquid ratios; (D): ultrasonic time and solid-to-liquid ratios; (E): ultrasonic temperature and incubation time; (F): water content and incubation time; (G): ultrasonic time and incubation time; (H): water content and ultrasonic temperature; (I): ultrasonic time and ultrasonic temperature; (J): ultrasonic time and water content; TPhC: Total phenolic content; TFlC: Total flavonoid content.

Three-dimensional response surface plots (Fig. 4A-J) were employed to explore the interactive effects of process parameters on the recovery of TPhC. As illustrated in Fig. 4B, TPhC in ECLP increased with the elevation of SLR and UTemp. When both SLR and UTemp were simultaneously increased, their combined effect also enhanced TPhC. Fig. 4A demonstrates that increasing SLR continued to influence TPhC positively. However, when SLR and IT were increased simultaneously, a diminishing impact on TPhC was observed. A similar trend was noted for the combination of UT and IT, their combined enhancement led to reduced extraction efficiency. Based on the model predictions, the optimal conditions for maximizing TPhC extraction from ECLP were SLR at 1:59 g/mL, WC at 12 %, UTemp at 43 °C, UT at 6.88 min, and IT at 6.33 min.

Concerning flavonoid extraction efficiency, the linear terms (P_2_) and quadratic terms (P_6_^2^) had statistically significant effects on TFlC (p < 0.05). As shown in equation (12), WC and the quadratic term of EA exhibited negative coefficients. Three-dimensional response surface plots (Fig. S4A-C) were utilized to investigate the interactive influences of the extraction parameters on TFlC recovery. Based on this interpretation and the statistical findings in Table S4, the interactions between SLR and WC, SLR and EA, WC and EA were determined to be non-significant. Accordingly, the optimal extraction conditions for maximizing TFlC from ECLP were identified as: SLR = 1:67 g/mL, WC = 14 %, EA = 1.28 U/g.

#### 3.5.2 Model validation

Validation experiments were performed under the optimal conditions of the UAE-EAE process to verify the accuracy of the polynomial regression models, as outlined in Table 3. The optimized parameters for extracting phenolics and flavonoids from ECLP were established. For TPhC, the optimal conditions included: SLR at 1:59 g/mL, WC at 12 %, UTemp at 43 °C, UT at 6.88 min, and IT at 6.33 min. In the case of TFlC, the corresponding optimal parameters were: SLR at 1:67 g/mL, WC at 14 % and EA at 1.28 U/g. The prediction errors for TPhC and TFlC were 3.08 % and 9.55 %, respectively, indicating high model accuracy. These findings confirm the robustness of the regression models and their applicability for estimating TPhC and TFlC yields from ECLP within the experimental range studied.

**Table 3:** Predicted results and experimental results of TPhC and TFlC at the optimal conditions.

Measurement	Conditions	Predicted values	Experimental values	Prediction error (%)
TPhC (mg GAE/g)	SLR: 1/59 (g/mL) WC: 12 (%) UTemp: 43 °C UT: 6.88 (min) IT: 6.33 (min)	35.29	36.41 ± 2.50	3.08
TFlC (mg QE/g)	SLR: 1/67 (g/mL) WC: 14 (%) EA: 1.28 U/g	16.95	18.74 ± 1.28	9.55

* TPhC: Total phenolic content; TFlC: Total flavonoid content; SLR: Solid-to-liquid ratio; WC: Water content; UTemp: Ultrasonic temperature; UT: Ultrasonic time; EA: Enzyme activity; IT: Incubation time.

#### 3.5.3 Antioxidant activity of extracts from the UAE-EAE process

The results in Table 4 demonstrate significant differences between the extraction methods in terms of antioxidant activities. Among the tested techniques, the combined UAE-EAE method exhibited the most significant antioxidant capacities for both TPhC (36.41 ± 2.50  mg GAE/g) and TFlC (18.74 ± 1.28  mg QE/g), indicating a pronounced synergistic effect between ultrasound and enzymatic treatment. This synergy enhances cell wall disruption and facilitates the cleavage of bonds between carbohydrates and phenolic compounds, thereby promoting the release of free phenolics with potent antioxidant properties [59,60]. Furthermore, it has been reported that NADES possess intrinsic antioxidant activity, which can contribute to the measured antioxidant capacity of extracts [61]. Antioxidant capacity strongly correlates with the content of antioxidant compounds, owing to their ability to donate electrons or hydrogen atoms to neutralize free radicals [62]. In conclusion, the UAE-EAE method emerges as the most effective technique for recovering phenolic and flavonoid compounds from ECLP. This evidence suggests its potential as a robust and sustainable approach for producing antioxidant-rich extracts in food, cosmetic, or pharmaceutical formulations.

**Table 4:** Antioxidant activities of ECLP extracts from four methods.

	Methods/Criteria	Bioactive compounds	DPPH (μM TE/g)	ABTS (μM TE/g)	FRAP (mg TE/g)
TPhC	UAE-EAE	36.41 ± 2.50a	29.7 ± 0.33a	624.51 ± 11.53a	96.07 ± 1.87a
	EAE	19.85 ± 1.23b	28.46 ± 0.55b	540.44 ± 12.82b	85.67 ± 3.57b
	UAE	19.74 ± 1.05b	25.2 ± 0.48c	503.43 ± 15.18c	82.55 ± 1.58b
	NAE	15.68 ± 0.94c	22.46 ± 0.3d	452.25 ± 9.07d	69.93 ± 2.98c
TFlC	UAE-EAE	18.74 ± 1.28a	17.74 ± 0.38a	215.49 ± 8.77a	31.13 ± 1.5a
	EAE	15.63 ± 0.87b	15.18 ± 0.57b	105 ± 22.09b	31.51 ± 0.85a
	UAE	14.45 ± 1.16b	15.66 ± 0.94b	122.5 ± 19.64b	23.14 ± 1.1b
	NAE	10.27 ± 1.34c	10.07 ± 0.57c	50.15 ± 8.3c	18.43 ± 0.93c

* TPhC: Total phenolic content; TFlC: Total flavonoid content; UAE: ultrasonic- assisted extraction technique; EAE: enzymatic-assisted extraction technique UAE-EAE: ultrasonic-enzymatic-assisted extraction technique; NAE: None-Assisted Extraction.

### 3.6 Extraction kinetic models of NADES-based UAE-EAE

**Fig. 5:**
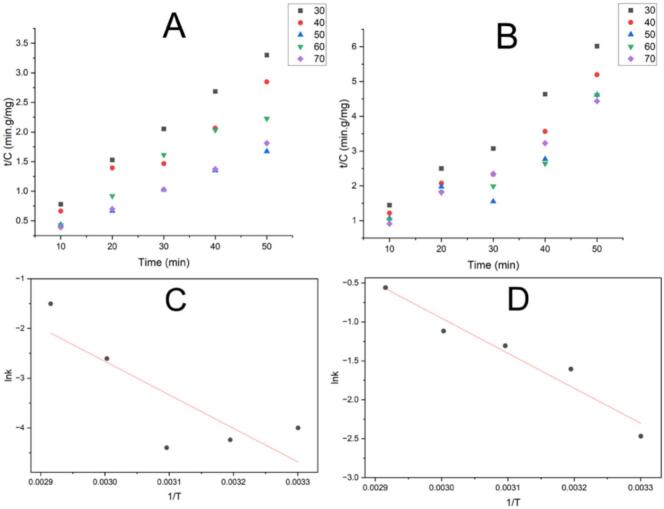
Changes in t/Ct values over time during the UAE-EAE process; (A): Time-dependent t/Ct variation for total phenolic content (TPhC); (B): Time-dependent t/Ct variation for total flavonoid content (TFlC); (C): Arrhenius plot derived from the second-order kinetic model for TPhC; (D): Arrhenius plot based on the second-order kinetic model for TFlC.

The study applied kinetic modelling to describe how phenolics and flavonoids are extracted using the NADES-based UAE-EAE process. The goal was to support future industrial applications. The experiments were conducted under constant conditions, varying the incubation temperature (30, 40, 50, 60, and 70 °C) and incubation time (10, 20, 30, 40, and 50  min). Fig. 5A-B shows the second-order kinetic model of the UAE-EAE process for extracting TPhC and TFlC, and kinetic model parameters are detailed in Table 5. The extraction rate, represented by the initial rate constant (h), increased as the temperature rose. This rise suggests that higher temperatures improve both solvent diffusion and facilitate the release of compounds from plant cells. A similar observation was reported in previous research on bioactive compound recovery from *Vernonia cinerea* using microwave-assisted hydro-distillation and Soxhlet extraction [63]. Arrhenius plots (Fig. 5C and 5D) were used to investigate the extraction mechanism further. These plots show the natural logarithm of the rate constant (ln k) versus the reciprocal of temperature (1/T). From this, the activation energy (E_a_) of the extraction process for TPhC and TFlC was calculated. If E_a_ < 20  kJ/mol, diffusion controls the process; between 20 and 40  kJ/mol, diffusion and solubilization are involved; and if E_a_ > 40  kJ/mol, solubilization dominates [64]. The E_a_ for phenolic extraction was 55.95  kJ/mol, suggesting a solubilization mechanism. For flavonoid extraction, E_a_ was 37.35  kJ/mol, indicating a combined effect of diffusion and solubilization. Vo et al. applied a second-order kinetic approach to investigate the recovery process of bioactive compounds from *Codonopsis pilosula* using NADES-assisted ultrasonic-microwave extraction (UMAE). Their findings indicated that the release of flavonoids followed a diffusion-driven mechanism, whereas the extraction of phenolic compounds was influenced by both diffusion and solubilization processes [65].

**Table 5:** Second-order kinetic parameters for the UAE-EAE extraction of TPhC and TFlC from ECLP.

T(⁰C)	$c_{eq}\left(\frac{mg}{g}\right)$	$h(\frac{mg}{g∙min})$	$k(\frac{g}{g∙min})$	$a(\frac{g}{mg})$	$b(\frac{g}{mg})$	$R_{1}^{2}$	$E_{a}\left(\frac{kj}{mol}\right)$	lnAe	$R_{2}^{2}$
TPhC									
30	16.13	4.77	0.0183	0.062	0.2097	0.9976			
40	19.84	5.68	0.0144	0.0504	0.1762	0.9501			
50	31.65	12.33	0.0123	0.0316	0.0811	0.9963	55.95	17.54	0.6687
60	21.23	33.33	0.0739	0.0471	0.03	0.9666			
70	28.33	178.57	0.2225	0.0353	0.0056	0.9953			
TFlC									
30	8.87	6.66	0.0848	0.1128	0.1501	0.9769			
40	10.59	22.52	0.2007	0.0944	0.0444	0.9345			
50	12.64	43.29	0.2709	0.0791	0.0231	0.8072	37.35	12.53	0.9465
60	12.52	51.28	0.3274	0.0799	0.0195	0.8706			
70	11.83	80.00	0.5712	0.0845	0.0125	0.9832			

* TPhC: Total phenolic content; TFlC: Total flavonoid content; c_eq_: the concentration of phenolics or flavonoids at the equilibrium point; h: the initial extraction rate; k: the second-order extraction rate constant; $E_{a}$: activation energy.

### 3.7 Structural surface analysis and crystallinity assessment of ECLP

**Fig. 6:**
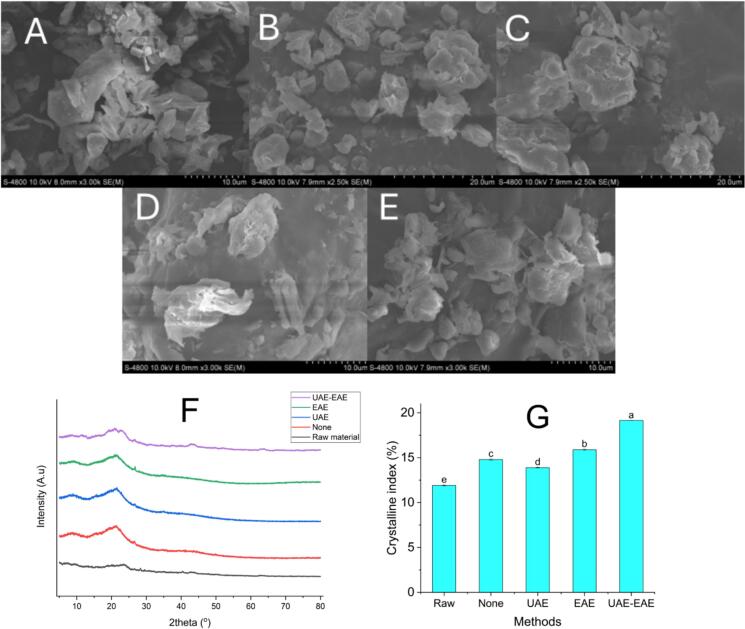
Comparative analysis of surface morphology and X-ray diffraction patterns between untreated and NADES-treated ECLP; (A): SEM image of raw ECLP; (B): SEM image of untreated ECLP; (C): SEM image of ECLP treated with UAE; (D): SEM image of ECLP treated with EAE; (E): SEM image of ECLP treated with UAE-EAE; (F): X-ray diffraction of ECLP from 5 methods; (G): Cellulose crystallinity index (CCI) values of untreated and NADES-treated ECLP samples (Different letters (a, b, c, d, e) indicate statistically significant differences). UAE: ultrasonic-assisted extraction technique; EAE: enzymatic-assisted extraction technique; UAE-EAE: ultrasonic-enzymatic-assisted extraction technique.

SEM was utilized to investigate the surface morphology of ECLP. Fig. 6A-E demonstrated the surface of ECLP under various extraction techniques, including raw powder (A), non-assisted extraction (NAE, B), ultrasound-assisted extraction (UAE, C), enzyme-assisted extraction (EAE, D), and the combined UAE-EAE approach (E). The raw sample and the NAE-treated sample displayed relatively intact and smooth surfaces. It is due to the passive soaking method exerting minimal influence on the plant cell architecture. The UAE-treated sample revealed surface damage marked by the emergence of pores. These microstructural features are indicative of the ultrasonic cavitation effect. In this process, collapsing bubbles generate localized microjets that break down cell walls and reorganize internal networks [66]. The EAE sample (Fig. 6D) exhibited surface roughening and partial cell wall collapse. This phenomenon was caused by the enzymatic degradation of amorphous cellulose regions, which weakened the intercellular binding forces [67]. The combined UAE-EAE condition (Fig. 6E) produced the most pronounced disruption. SEM images showed deep grooves, expanded pores, and disintegrated cell surfaces, reflecting enhanced permeability and matrix breakdown. This structural transformation is due to the synergy between ultrasonication and enzymatic hydrolysis. Ultrasound first opens the cell wall through cavitation. This destruction enables more effective enzyme infiltration, accelerating hydrolytic cleavage of internal polysaccharides and revealing cellulose microfibrils [68]. These findings are consistent with the observations by Li et al. [68], who documented similar cellular deconstruction patterns in *Armillaria mellea* using a combined ultrasonic-enzyme treatment approach.

XRD is a non-destructive analytical technique widely used to evaluate the crystalline structure of materials, particularly effective for detecting structural alterations at the molecular level. This study utilised XRD to assess the crystallinity changes in ECLP following treatment with the UAE-EAE method. The primary focus was on monitoring the CCI, a parameter reflective of structural disruption induced by the combined effects of NADES and ultrasonic-enzymatic forces. The diffraction patterns of both untreated and treated ECLP samples were recorded within the theta angle range of 5 to 80◦. Distinct peaks observed at 18.0 and 22.5 were assigned to the amorphous and crystalline phases of cellulose, respectively (Fig. 6F). The calculated CCI values for raw ECLP and NAE-treated samples (Fig. 6G) were 11.91 % and 14.80 %, respectively. CCI values for UAE, EAE, and UAE-EAE samples were recorded at 13.89 %, 15.88 %, and 19.14 %, respectively. An increasing trend in crystallinity was apparent after treatment, indicating the hydrolysis of the amorphous cellulose matrix. This increase is attributed to the ability of NADES to form hydrogen bonds with cellulose's amorphous regions [69]. In the UAE case, NADES is believed to interact with cellulose's amorphous regions. However, NADES does not fully degrade amorphous areas, resulting in partial solubilization without extensive breakdown. This limited impact leaves much of the crystalline cellulose and hemicellulose intact. EAE treatment primarily targets the amorphous cellulose. Cellulase enzymes cannot access the crystalline domains. Thus, the structural core is largely preserved. After elution, significant portions of the original cellulose structure remain, while minor fractions such as lignin are more readily removed [69]. The combined UAE-EAE approach amplifies enzymatic hydrolysis by creating mechanical fissures through ultrasonication, enabling better enzyme penetration. Simultaneously, NADES enhances structural loosening by solubilizing amorphous forms. As a result, the coordinated action facilitates deeper hydrolysis and removal of amorphous cellulose fragments. This observation is consistent with earlier findings by Kumar et al. [69], who demonstrated that NADES pre-treatment disrupted lignin structures in rice straw while leaving crystalline cellulose largely unaffected in the absence of enzymatic hydrolysis.

**Fig. 7:**
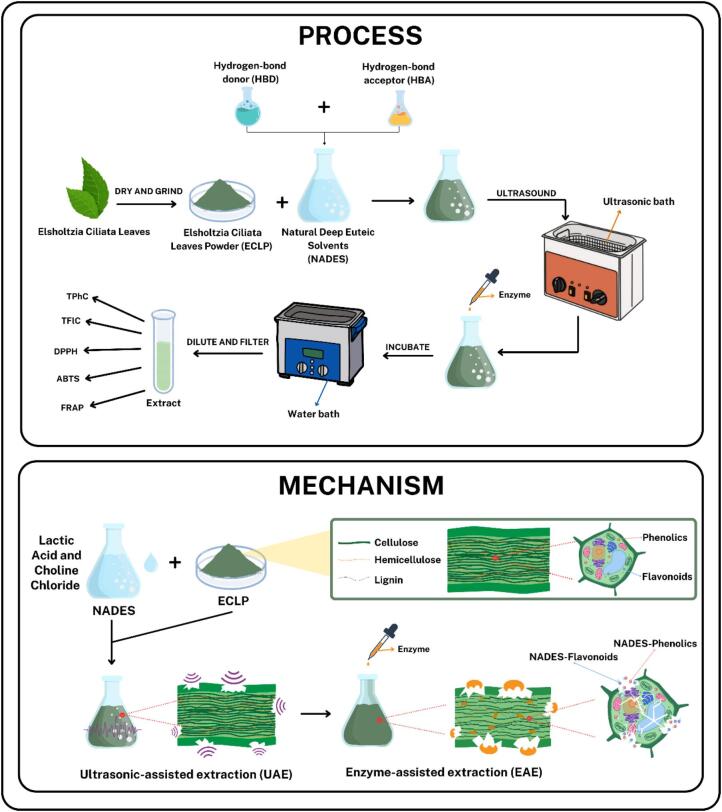
The procedure and mechanism of the NADES-based UAE-EAE process.

Based on the kinetics, SEM imaging, and XRD analysis, a comprehensive extraction mechanism underlying the UAE-EAE process can be inferred. The NADES-based UAE-EAE procedure and mechanism are presented in Fig. 7. The kinetic data quantify extraction efficiency and indicate that diffusion and solubilization contribute to the compound release mechanism. Structural modifications in the ECLP matrix are further evidenced by XRD, which reveals alterations in crystallinity through changes in the CCI. SEM micrographs offer visual confirmation of surface damage and material breakdown. The interaction between NADES and the ECLP surface initiates structural loosening by converting ordered crystalline cellulose into a more disordered amorphous form. Simultaneously, NADES can penetrate the lignocellulosic network and partially dissolve lignin, facilitating the separation of cellulose fibers. Ultrasonic cavitation intensifies this process by enhancing the accessibility of NADES to both lignin and crystalline domains. The resulting structural weakening fosters extensive cell wall disruption and fiber liberation. These changes create ideal pathways for enzymatic penetration, enabling enzymes to adhere directly to the exposed cellulose and perform hydrolysis effectively. As polysaccharide bonds are cleaved and cellular architecture is dismantled, phenolic and flavonoid compounds are released more from the plant matrix.

## 4 Conclusion

This research investigated the extraction of phenolics and flavonoids from *Elsholtzia ciliata* leaves (ECLP) using a green UAE-EAE method with NADES as the extraction medium. Among eight tested NADES formulations, Lac-Cho (lactic acid: choline chloride) exhibited the highest yield for phenolics and flavonoids. According to the Plackett-Burman design, SLR, UTemp, and IT significantly affected TPhC extraction, while SLR, WC, and EA influenced TFlC. The optimized extraction conditions for TPhC were SLR: 1:59 g/mL, WC: 12 %, UTemp: 43⁰C, UT: 6.88 min, and IT: 6.33 min, yielding 36.41 ± 2.50  mg GAE/g. The optimized extraction conditions for TFlC were SLR: 1:67 g/mL, WC: 14 %, EA: 1.28 U/g. Extraction kinetics followed a second-order model. The activation energy of phenolic extraction (E_a_ = 55.95 kJ/mol) confirmed a solubilization-controlled mechanism, while that of flavonoids (E_a_ = 37.35 kJ/mol) suggested a combined diffusion-solubilization mechanism. SEM and XRD analyses revealed surface damage and increased crystallinity of ECLP after treatment, supporting the efficacy of NADES-based UAE-EAE. This study demonstrates that combining NADES and UAE-EAE offers a sustainable and efficient platform for recovering bioactive compounds from Vietnamese balm, with potential applications in the nutraceutical and food industries. NADES can be directly applied to food products for several reasons. Their components are composed of organic acids and polyols such as monosaccharides. NADES are capable of maintaining the stability of natural compounds and the structural integrity of macromolecules such as proteins, owing to the inhibitory activity of microbial growth and an extensive hydrogen-bonding network. However, this study did not address the issue of NADES recyclability. The phenolic and flavonoid constituents in the extraction solutions were not analyzed due to equipment limitations and finances. Therefore, future studies could employ molecular dynamics simulations and HPLC analysis to elucidate the interactions between NADES and enzymes, as well as between NADES and bioactive compounds.

## Data availability

Data will be made available on request.

## Funding Declaration

There are no funding sources for the manuscript.

## CRediT authorship contribution statement

**Tan Phat Vo:** Writing – review & editing, Writing – original draft, Visualization, Software, Methodology, Investigation, Formal analysis, Data curation, Conceptualization. **Thi Hoang Trang Nguyen:** Writing – original draft, Investigation, Formal analysis. **Ha Bao Tran Nguyen:** Writing – original draft, Visualization. **Hoang Nhan Nguyen:** Writing – original draft, Visualization, Investigation. **Nguyen Van Nhi Le:** Visualization, Investigation, Formal analysis. **Minh Hoa Ha:** Investigation, Formal analysis. **Gia Bao Pham:** Writing – original draft, Visualization, Investigation. **Dinh Quan Nguyen:** Writing – original draft, Investigation, Formal analysis.

## Declaration of competing interest

The authors declare that they have no known competing financial interests or personal relationships that could have appeared to influence the work reported in this paper.
