# Supplementary material for: Enhancing phenolic and flavonoid recovery from Vietnamese balm using green solvent-based ultrasonic-enzymatic-assisted extraction
Source: Ultrason Sonochem. 2025 Sep 1;121:107546. doi: 10.1016/j.ultsonch.2025.107546 (PMC12444158; doi:10.1016/j.ultsonch.2025.107546)
Supplement: Supplementary Data 1 [file mmc1.docx]

**Enhancing the phenolic and flavonoid recovery from** **Vietnamese balm using green solvents**

Tan Phat Vo^1,2^, Thi Hoang Trang Nguyen^2,3^, Ha Bao Tran Nguyen^1,2^, Hoang Nhan Nguyen^1,2^, Nguyen Van Nhi Le^1,2^, Minh Hoa Ha^1,2^, Gia Bao Pham^1,2^, Dinh Quan Nguyen^1,2*^

^1^Laboratory of Biofuel and Biomass Research, Faculty of Chemical Engineering, Ho Chi Minh City University of Technology (HCMUT), 268 Ly Thuong Kiet Street, District 10, Ho Chi Minh City, Vietnam

^2^Vietnam National University Ho Chi Minh City, Linh Trung Ward, Thu Duc City, Ho Chi Minh City, Vietnam

^3^School of Chemical and Environmental Engineering, International University (HCMIU), Linh Trung Ward, Thu Duc City, Ho Chi Minh City, Vietnam

^*^Corresponding author: [ndquan@hcmut.edu.vn](mailto:ndquan@hcmut.edu.vn) (Dinh Quan Nguyen)


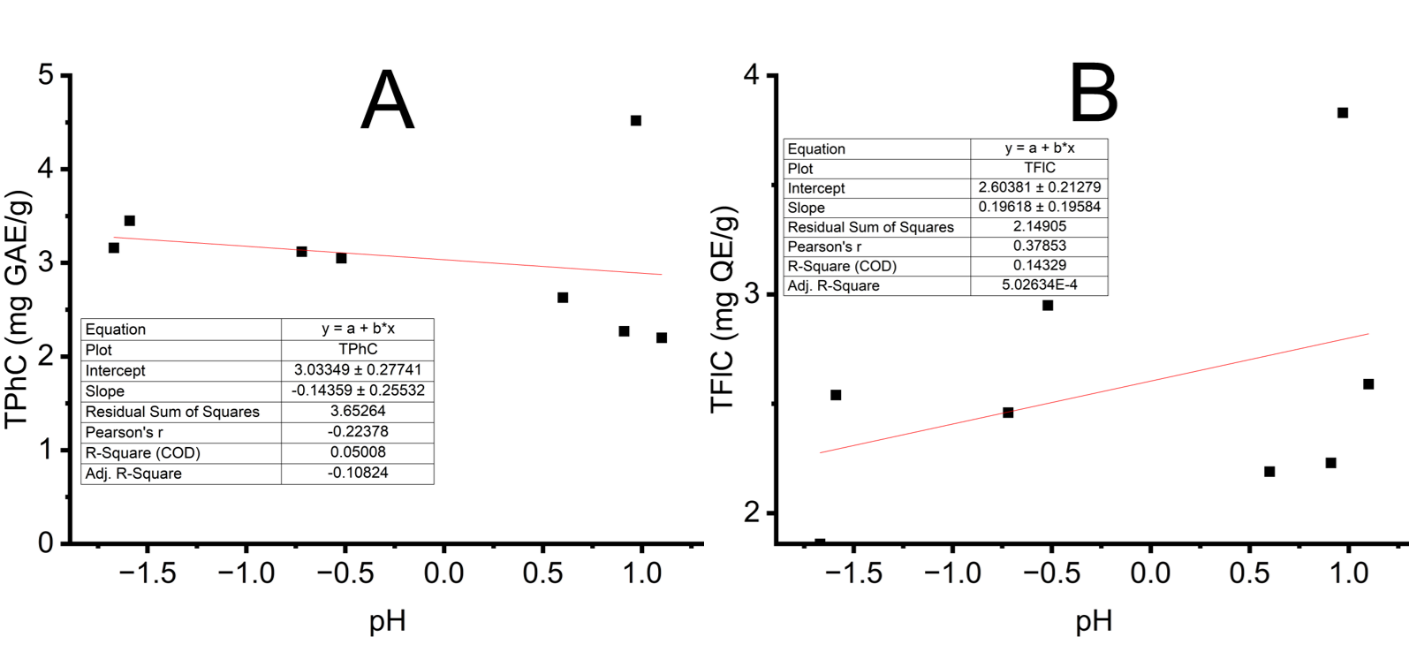


Figure S1 The correlation between the pH levels of NADES and the extraction efficiency of bioactive compounds; (A): TPhC; (B): TFlC; TPhC: Total phenolic content; TFlC: Total flavonoid content


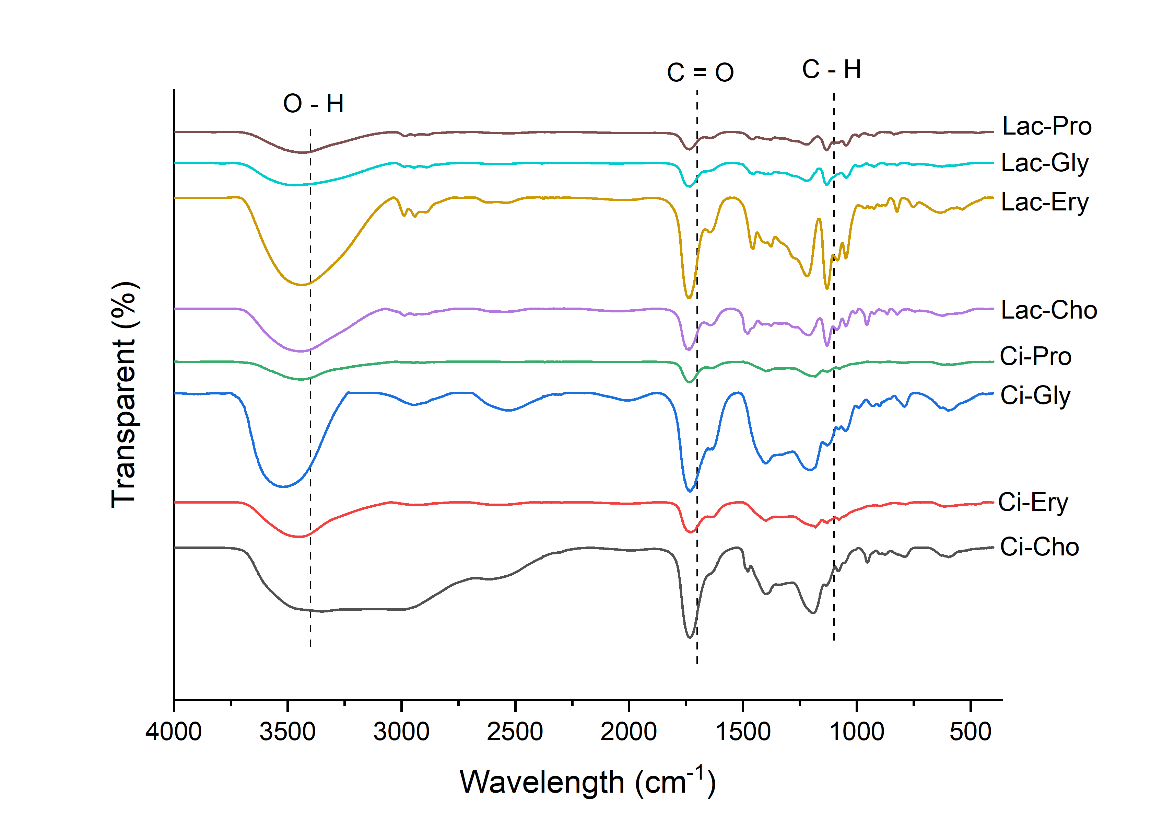


Figure S2 FTIR spectrum of eight NADESs


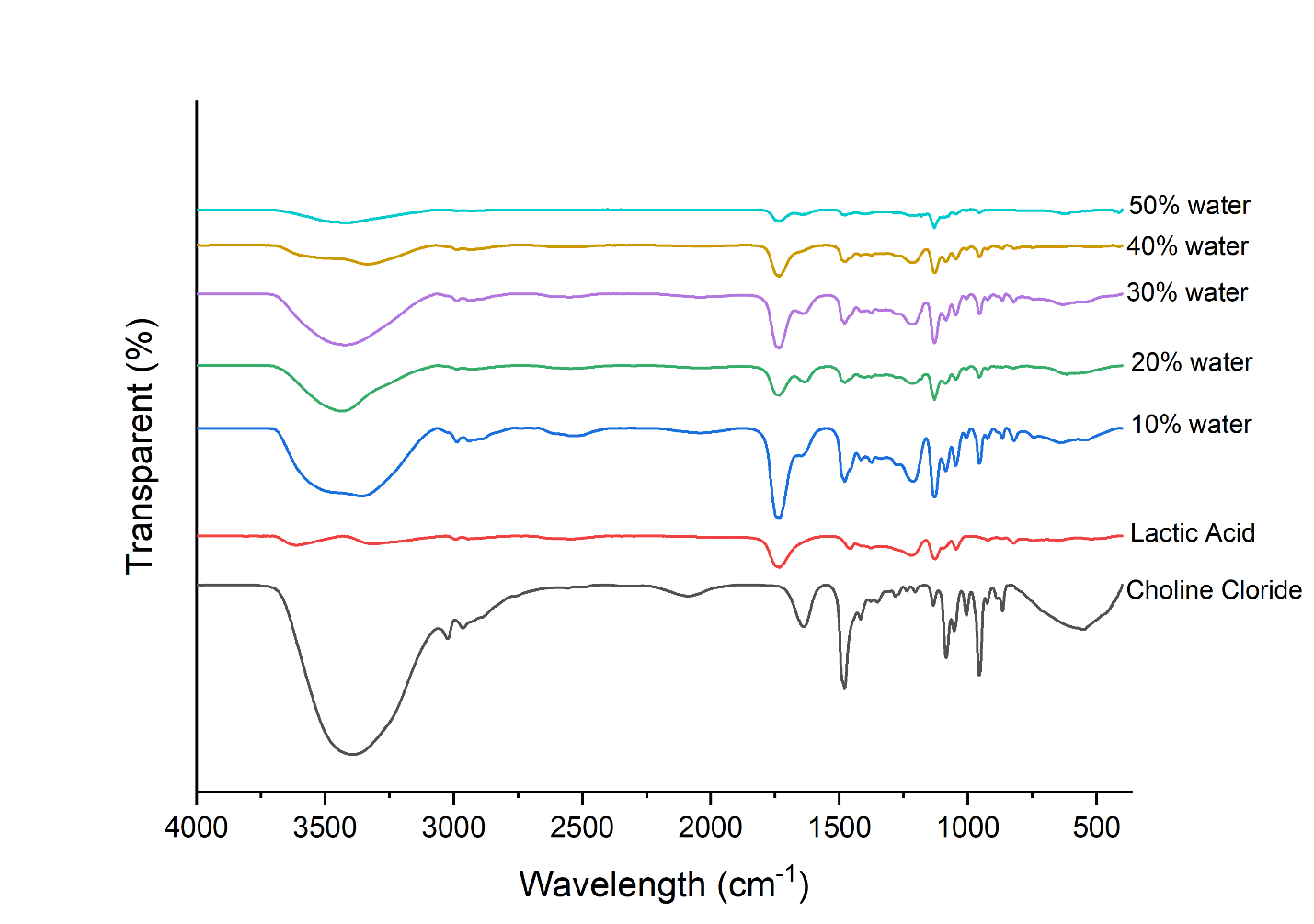


Figure S3 FTIR spectrum of various water content in NADES Lac-Cho


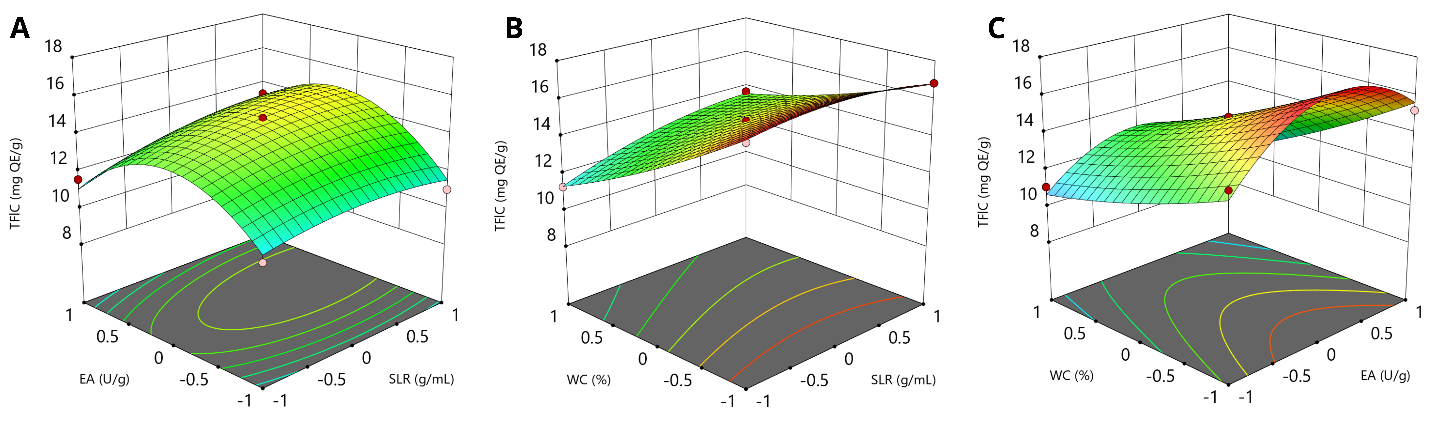


Figure S4 Three-dimensional response surface plots illustrating the interactive effects of UAE-EAE process on TFlC; (A): enzyme and solid-to-liquid ratios; (B): water content and solid-to-liquid ratios; (C): water content and enzyme activity; TPhC: Total phenolic content; TFlC: Total flavonoid content.

Table S1 The experimental results of Plackett–Burman Design

| SLR | WC | UP | Utemp | UT | EA | IT | TPhC | TFlC |
| --- | --- | --- | --- | --- | --- | --- | --- | --- |
| 1 | 1 | -1 | 1 | 1 | -1 | 1 | 19.14 | 8.05 |
| 1 | -1 | 1 | -1 | -1 | -1 | 1 | 19.73 | 7.25 |
| 1 | 1 | -1 | 1 | -1 | -1 | -1 | 17.4 | 8.10 |
| -1 | -1 | 1 | 1 | 1 | -1 | 1 | 27.31 | 11.29 |
| -1 | -1 | -1 | 1 | 1 | 1 | -1 | 21.53 | 10.16 |
| -1 | 1 | -1 | -1 | -1 | 1 | 1 | 24.83 | 11.27 |
| 1 | 1 | 1 | -1 | 1 | 1 | -1 | 10.98 | 5.52 |
| 1 | -1 | -1 | -1 | 1 | 1 | 1 | 19.59 | 5.02 |
| -1 | -1 | -1 | -1 | -1 | -1 | -1 | 21.02 | 10.46 |
| -1 | 1 | 1 | -1 | 1 | -1 | -1 | 17.74 | 12.05 |
| 1 | -1 | 1 | 1 | -1 | 1 | -1 | 17.52 | 6.86 |
| -1 | 1 | 1 | 1 | -1 | 1 | 1 | 27.46 | 12.09 |

* TPhC: Total phenolic content; TFlC: Total flavonoid content; SLR: Solid-to-liquid ratio; WC: Water content; UP: Ultrasonic power; Utemp: Ultrasonic temperature; UT: Ultrasonic time; EA: Enzyme activity; IT: Incubation time.

Table S2 Statistical evaluation of the Plackett–Burman Design experiments through ANOVA

| Term |  | TPhC |  |  | TFlC |  |  |
| --- | --- | --- | --- | --- | --- | --- | --- |
|  |  | Coef | T-Value | P-Value | Coef | T-Value | P-Value |
| Constant | P_0_ | 20.354 | 78.51 | <0.001 | 9.01 | 59.28 | <0.001 |
| SLR | P_1_ | -2.961 | -11.42 | <0.001 | -2.21 | -14.54 | <0.001 |
| WC | P_2_ | -0.762 | -2.94 | 0.042 | 0.503 | 3.31 | 0.03 |
| UP | P_3_ | -0.231 | -0.89 | 0.424 | 0.168 | 1.1 | 0.331 |
| UTemp | P_4_ | 1.373 | 5.29 | 0.006 | 0.415 | 2.73 | 0.052 |
| UT | P_5_ | -0.972 | -3.75 | 0.02 | -0.328 | -2.16 | 0.097 |
| EA | P_6_ | -0.036 | -0.14 | 0.897 | -0.524 | -3.45 | 0.026 |
| IT | P_7_ | 2.656 | 10.24 | 0.001 | 0.153 | 1.01 | 0.371 |
| R^2^ (%) |  | 98.63 |  |  | 98.42 |  |  |
| Adjusted R^2^ (%) |  | 96.22 |  |  | 95.65 |  |  |
| Predicted R^2^ (%) |  | 87.63 |  |  | 85.75 |  |  |

* TPhC: Total phenolic content; TFlC: Total flavonoid content; SLR: Solid-to-liquid ratio; WC: Water content; UP: Ultrasonic power; Utemp: Ultrasonic temperature; UT: Ultrasonic time; EA: Enzyme activity; IT: Incubation time.

Table S3 The experimental results of Box–Behnken Design

|  | SLR | IT | Utemp | WC | UT | TPC |  | SLR | EA | WC | TFC |
| --- | --- | --- | --- | --- | --- | --- | --- | --- | --- | --- | --- |
| 1 | 0 | 0 | 1 | 0 | 1 | 21.06 | 1 | 0 | 1 | 1 | 9.55 |
| 2 | 0 | -1 | 1 | 0 | 0 | 24.13 | 2 | -1 | 0 | 1 | 11.29 |
| 3 | -1 | 0 | 0 | -1 | 0 | 18.50 | 3 | 1 | 1 | 0 | 13.38 |
| 4 | 0 | 0 | 0 | -1 | -1 | 22.34 | 4 | -1 | -1 | 0 | 11.00 |
| 5 | -1 | 0 | 0 | 1 | 0 | 18.93 | 5 | 0 | -1 | -1 | 14.38 |
| 6 | 0 | 0 | 0 | -1 | 1 | 21.77 | 6 | -1 | 0 | -1 | 16.80 |
| 7 | 1 | 0 | 0 | 1 | 0 | 25.53 | 7 | 0 | 1 | -1 | 15.21 |
| 8 | 1 | 0 | 1 | 0 | 0 | 33.92 | 8 | -1 | 1 | 0 | 11.57 |
| 9 | 0 | 1 | 0 | -1 | 0 | 25.20 | 9 | 0 | -1 | 1 | 11.07 |
| 10 | -1 | 1 | 0 | 0 | 0 | 22.22 | 10 | 1 | -1 | 0 | 11.02 |
| 11 | 0 | 1 | 1 | 0 | 0 | 23.14 | 11 | 0 | 0 | 0 | 14.86 |
| 12 | 0 | 1 | 0 | 1 | 0 | 18.90 | 12 | 1 | 0 | -1 | 16.84 |
| 13 | -1 | 0 | 0 | 0 | -1 | 17.25 | 13 | 1 | 0 | 1 | 13.69 |
| 14 | 0 | 0 | 1 | -1 | 0 | 25.46 | 14 | 0 | 0 | 0 | 14.86 |
| 15 | 0 | 0 | -1 | 0 | 1 | 19.52 |  |  |  |  |  |
| 16 | 1 | 1 | 0 | 0 | 0 | 23.45 |  |  |  |  |  |
| 17 | 1 | 0 | -1 | 0 | 0 | 23.36 |  |  |  |  |  |
| 18 | -1 | 0 | -1 | 0 | 0 | 21.49 |  |  |  |  |  |
| 19 | 1 | 0 | 0 | -1 | 0 | 29.30 |  |  |  |  |  |
| 20 | 0 | 0 | -1 | 0 | -1 | 23.75 |  |  |  |  |  |
| 21 | 0 | -1 | 0 | 0 | -1 | 21.12 |  |  |  |  |  |
| 22 | 0 | -1 | 0 | 1 | 0 | 21.18 |  |  |  |  |  |
| 23 | 1 | -1 | 0 | 0 | 0 | 34.00 |  |  |  |  |  |
| 24 | 0 | 1 | 0 | 0 | 1 | 19.88 |  |  |  |  |  |
| 25 | 0 | -1 | -1 | 0 | 0 | 24.40 |  |  |  |  |  |
| 26 | 0 | 1 | -1 | 0 | 0 | 22.93 |  |  |  |  |  |
| 27 | -1 | 0 | 0 | 0 | 1 | 17.63 |  |  |  |  |  |
| 28 | 0 | 0 | 0 | 1 | 1 | 17.30 |  |  |  |  |  |
| 29 | 0 | 0 | 1 | 1 | 0 | 22.38 |  |  |  |  |  |
| 30 | -1 | -1 | 0 | 0 | 0 | 15.30 |  |  |  |  |  |
| 31 | 0 | 0 | 0 | 0 | 0 | 34.45 |  |  |  |  |  |
| 32 | 0 | 0 | -1 | 1 | 0 | 21.55 |  |  |  |  |  |
| 33 | 1 | 0 | 0 | 0 | -1 | 30.22 |  |  |  |  |  |
| 34 | 0 | -1 | 0 | 0 | 1 | 21.09 |  |  |  |  |  |
| 35 | 0 | 0 | -1 | -1 | 0 | 23.90 |  |  |  |  |  |
| 36 | 0 | -1 | 0 | -1 | 0 | 23.68 |  |  |  |  |  |
| 37 | 0 | 0 | 0 | 1 | -1 | 20.35 |  |  |  |  |  |
| 38 | 0 | 0 | 0 | 0 | 0 | 34.45 |  |  |  |  |  |
| 39 | 1 | 0 | 0 | 0 | 1 | 26.84 |  |  |  |  |  |
| 40 | -1 | 0 | 1 | 0 | 0 | 18.71 |  |  |  |  |  |
| 41 | 0 | 0 | 1 | 0 | -1 | 24.42 |  |  |  |  |  |
| 42 | 0 | 1 | 0 | 0 | -1 | 25.52 |  |  |  |  |  |

SLR: Solid-to-liquid ratio; WC: Water content; Utemp: Ultrasonic temperature; UT: Ultrasonic time; EA: Enzyme activity; IT: Incubation time.

Table S4 ANOVA results corresponding to the optimization experiments based on the Box–Behnken design.

| TPhC | | | | TFlC | | | |
| --- | --- | --- | --- | --- | --- | --- | --- |
| Factors | Coefficient | F-value | P-value | Factors | Coefficient | F-value | P-value |
| P_0_ | 34.45 | 31.77 | < 0.0001 | P_0_ | 14.86 | 18.16 | 0.007 |
| P_1_ | 4.79 | 264.21 | < 0.0001 | P_1_ | 0.533 | 5.35 | 0.08 |
| P_2_ | -1.50 | 25.96 | < 0.0001 | P_2_ | -2.20 | 91.41 | 0.0007 |
| P_4_ | 0.77 | 6.85 | 0.0161 | P_6_ | 0.28 | 1.47 | 0.30 |
| P_5_ | -1.24 | 17.78 | 0.0004 | P_1_P_6_ | 0.45 | 1.89 | 0.24 |
| P_7_ | -0.23 | 0.60 | 0.4458 | P_1_P_2_ | 0.59 | 3.28 | 0.14 |
| P_1_P_7_ | -4.37 | 54.96 | < 0.0001 | P_2_P_6_ | -0.59 | 3.23 | 0.15 |
| P_1_P_4_ | 3.33 | 32.04 | < 0.0001 | P_1_² | -0.51 | 1.96 | 0.23 |
| P_1_P_2_ | -1.05 | 3.17 | 0.0893 | P_2_² | 0.30 | 0.6974 | 0.45 |
| P_1_P_5_ | -0.94 | 2.56 | 0.1247 | P_6_² | -2.61 | 51.33 | 0.002 |
| P_7_P_4_ | 0.12 | 0.04 | 0.842 |  |  |  |  |
| P_7_P_2_ | -0.95 | 2.60 | 0.1218 |  |  |  |  |
| P_7_P_5_ | -1.40 | 5.67 | 0.0267 |  |  |  |  |
| P_4_P_2_ | -0.18 | 0.10 | 0.7592 |  |  |  |  |
| P_4_P_5_ | 0.22 | 0.14 | 0.7165 |  |  |  |  |
| P_2_P_5_ | -0.62 | 1.11 | 0.303 |  |  |  |  |
| P_1_² | -4.83 | 84.84 | < 0.0001 |  |  |  |  |
| P_2_² | -6.53 | 155.02 | < 0.0001 |  |  |  |  |
| P_4_² | -5.04 | 92.40 | < 0.0001 |  |  |  |  |
| P_5_² | -7.04 | 180.53 | < 0.0001 |  |  |  |  |
| P_7_² | -5.70 | 118.36 | < 0.0001 |  |  |  |  |
| R² (%) | 96.80 |  |  |  | 97.61 |  |  |
| Adjusted R² (%) | 93.75 |  |  |  | 92.24 |  |  |
| Predicted R² (%) | 87.20 |  |  |  | 61.78 |  |  |

* TPhC: Total phenolic content; TFlC: Total flavonoid content
